# Identification of Cancer–associated metabolic vulnerabilities by modeling multi-objective optimality in metabolism

**DOI:** 10.1186/s12964-019-0439-y

**Published:** 2019-10-10

**Authors:** Ziwei Dai, Shiyu Yang, Liyan Xu, Hongrong Hu, Kun Liao, Jianghuang Wang, Qian Wang, Shuaishi Gao, Bo Li, Luhua Lai

**Affiliations:** 1 Center for Quantitative Biology, Academy for Advanced Interdisciplinary Studies, Peking University, Beijing, 100871 China; 2 Program of Cancer Research, Affiliated Guangzhou Women and Children’s Hospital, Zhongshan School of Medicine, Sun Yat-Sen University, Guangzhou, 510080 China; 3 Beijing National Laboratory for Molecular Sciences, State Key Laboratory for Structural Chemistry of Unstable and Stable Species, College of Chemistry and Molecular Engineering, Peking University, Beijing, 100871 China; 4 Peking-Tsinghua Center for Life Sciences, Peking University, Beijing, 100871 China

**Keywords:** Cancer metabolism, Drug discovery, Genome-scale metabolic model, Flux balance analysis, Pareto optimality

## Abstract

**Background:**

Cancer cells undergo global reprogramming of cellular metabolism to satisfy demands of energy and biomass during proliferation and metastasis. Computational modeling of genome-scale metabolic models is an effective approach for designing new therapeutics targeting dysregulated cancer metabolism by identifying metabolic enzymes crucial for satisfying metabolic goals of cancer cells, but nearly all previous studies neglect the existence of metabolic demands other than biomass synthesis and trade-offs between these contradicting metabolic demands. It is thus necessary to develop computational models covering multiple metabolic objectives to study cancer metabolism and identify novel metabolic targets.

**Methods:**

We developed a multi-objective optimization model for cancer cell metabolism at genome-scale and an integrated, data-driven workflow for analyzing the Pareto optimality of this model in achieving multiple metabolic goals and identifying metabolic enzymes crucial for maintaining cancer-associated metabolic phenotypes. Using this workflow, we constructed cell line-specific models for a panel of cancer cell lines and identified lists of metabolic targets promoting or suppressing cancer cell proliferation or the Warburg Effect. The targets were then validated using knockdown and over-expression experiments in cultured cancer cell lines.

**Results:**

We found that the multi-objective optimization model correctly predicted phenotypes including cell growth rates, essentiality of metabolic genes and cell line specific sensitivities to metabolic perturbations. To our surprise, metabolic enzymes promoting proliferation substantially overlapped with those suppressing the Warburg Effect, suggesting that simply targeting the overlapping enzymes may lead to complicated outcomes. We also identified lists of metabolic enzymes important for maintaining rapid proliferation or high Warburg Effect while having little effect on the other. The importance of these enzymes in cancer metabolism predicted by the model was validated by their association with cancer patient survival and knockdown and overexpression experiments in a variety of cancer cell lines.

**Conclusions:**

These results confirm this multi-objective optimization model as a novel and effective approach for studying trade-off between metabolic demands of cancer cells and identifying cancer-associated metabolic vulnerabilities, and suggest novel metabolic targets for cancer treatment.

**Graphical abstract:**

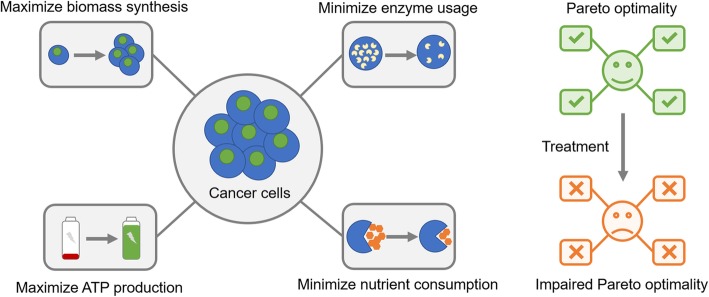

## Background

Metabolic reprogramming is recognized as an emerging hallmark of cancer [1–4]. Besides the “wasteful” metabolism known as aerobic glycolysis or the Warburg effect that was discovered almost a hundred years ago by Otto Warburg [5, 6], metabolism of malignant cells is shifted at the systematic level due to numerous factors including nutrient and oxygen availability in the tumor microenvironment, material and energy demands for rapid cell proliferation, and dysregulated signal transductions in malignant cells [7]. Targeting metabolic reprogramming in cancer is hence a promising strategy for designing highly selective anti-tumor therapeutics with several successful examples [8–11]. However, the human metabolic network covers thousands of enzymes, metabolites and crosstalks, which are also highly context dependent. The extreme complexity of human metabolic network greatly limits our ability to efficiently and accurately identify metabolic enzymes that serve as potential anti-tumor targets.

Genome-scale metabolic model (GSMM) is a powerful computational tool for studying metabolism [12–15] and has enabled researchers to elucidate the plausible mechanism of cancer-associated metabolic features such as the Warburg effect [16], quantify efficacies and side effects of cancer therapeutics [17–21], and unravel context-dependent functionality of metabolic enzymes during tumor progression [22–25]. Among various strategies, flux balance analysis (FBA) exhibits itself as a highly effective approach to analyze GSMMs [26]. FBA commonly assumes that cells optimize certain objective function by coordinating metabolic fluxes subjected to upper/ lower limits and stoichiometric constraints, by which both input and output fluxes are balanced to maintain the steady state at the systemic level. In particular, the assumption of maximized biomass production (representing for optimal cancer cell growth) has been widely used in previous studies modeling cancer metabolism [14, 15].

Despite the wide application of FBA-based computational methods, their fundamental assumption– maximization of growth rate in cancer cells – is still open to doubt. Although studies investigating the metabolic objectives of cancer cells were scarce, several studies focusing on unicellular organisms provided useful insights [27–29]. Interestingly, the hypothesis of single-objective metabolic optimization was challenged even in *Escherichia coli* which is significantly less complicated than eukaryotes. Comparison of experimentally-measured metabolic fluxes and the Pareto-optimal surface defined by multiple metabolic objectives revealed that cellular metabolism may be determined by trade-off among three competing objectives: maximization of biomass yield, maximization of ATP production, and minimization of gross metabolic fluxes [30]. Similarly, the trade-off between biomass yield and ATP production was also considered as one plausible mechanism underlying tumor-associated metabolic disorders [31].

In line with these findings, we present here a theoretical strategy involving multi-objective optimality for modeling cancer metabolism. Specifically, we developed algorithms for sampling balanced flux configurations with Pareto optimality and building individualized models based on multi-omics datasets. To demonstrate our methodology, we construct cell line-specific models for NCI-60 cancer cell lines and predict the impact of metabolic gene ablation on Pareto optimality, metabolism, and cell viability. With this approach, we identify several groups of metabolic enzymes essential for cell proliferation or the Warburg effect, and further validate these putative targets through survival analysis and cell-based experiments. These results will likely improve our understanding of cancer-associated metabolic disorders and reveal potential targets for novel cancer therapeutics.

## Methods

### Mathematical model and statistical analysis

The mathematical models and algorithms used in this study are explained in detail in Supplementary Methods. Statistical tests were performed using MATLAB. Algorithms for sampling the Pareto surface, constructing individualized models, and identifying targets were implemented in MATLAB code. For multiple hypothesis testing, *p*-values were corrected using the Benjamini-Hochberg procedure.

### Experimental validation of identified metabolic targets

For the validation of proliferation-promoting enzymes, cell lines were selected from the NCI-60 panel based on their predicted changes of biomass production flux using the NCI-60 individualized models as previously constructed. The simulation of enzymatic perturbations was performed using minimization of metabolic adjustment (MOMA) [32]. Cell lines predicted to have significant reduction of biomass production flux were selected for further experimental validation. For the validation of proliferation-suppressing and Warburg effect-suppressing enzymes, cell lines were selected to cover a range of different tissues of origin.

#### Cell culture

BT549, MDA_MB_231, A549, U87, SW_620, COLO205, and RPMI_8226 cell lines were purchased from the China Infrastructure of Cell Line Resources and cultured in RPMI containing 10% FBS and antibiotics. Purchased U251 and HeLa cells were cultured in DMEM containing 10% FBS. All cell lines were confirmed to be mycoplasma negative. shRNA constructs were transfected into cells using Lipofectamine and selected with corresponding antibiotics.

#### Immunoblot analysis

Cells were lysed with lysis buffer (25 mM Tris, 100 mM NaCl, 1% Triton X-100, 1 mM EDTA, 1 mM DTT, 1 mM NaVO_4_, 1 mM b-glycerol phosphate, and 1 mg/mL aprotinin), and then the lysates were resolved by SDS-PAGE and proteins transferred to PDVF membranes. The filters were incubated with various primary antibodies diluted in TBST (20 mM Tris, 135 mM NaCl, and 0.02% Tween 20). The primary antibodies were detected with horseradish peroxidase-conjugated secondary antibodies followed by exposure to ECL reagent.

#### Cell growth and metabolic assays

Cells were plated in dishes at a density of 5 × 10^4^ cells/dish and cultured in low serum medium for 5 consecutive days. Every other day one set of cells was collected and counted, while the medium on the remaining sets of cells was replenished. The oxygen consumption rate (OCR) and extracellular acidification rate (ECAR) were determined using a Seahorse XFe96 Analyzer (Agilent Technologies, Inc) by following the manufacturer’s protocol.

## Results

### Four-objective optimization model for cancer metabolism

**Fig. 1:**
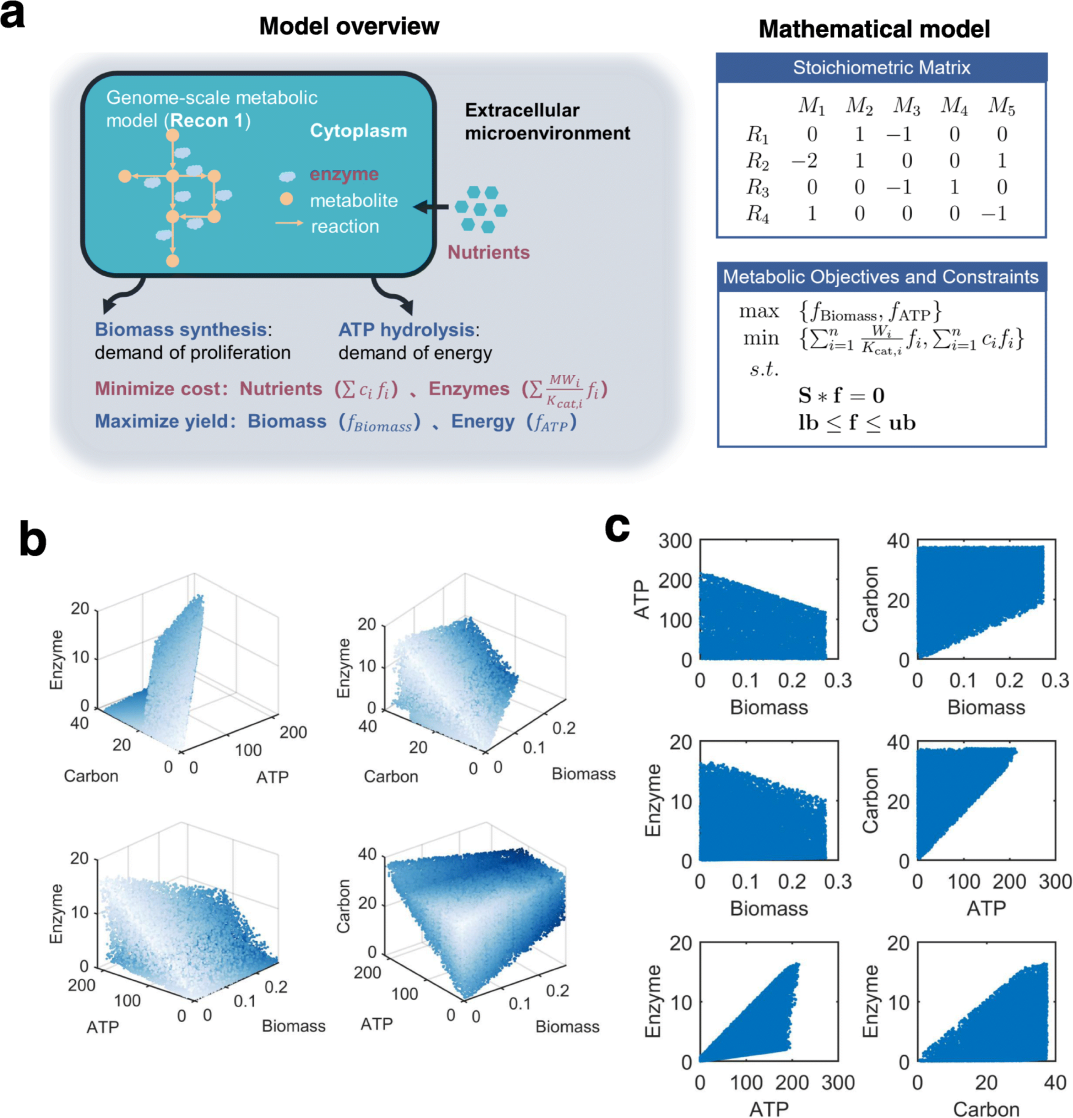
Four-objective optimization model for cancer metabolism. (**a**) Illustration of the four metabolic objectives incorporated in this model and mathematical description of its components. (**b**) The sampled Pareto surface projected on four ternary combinations of included objectives. Data points are presented in shade to depict the shape of Pareto surface. (**c**) Distributions of values for binary combinations of objectives in the sampled Pareto solutions

To develop a multi-objective optimization model for cancer metabolism, we hypothesized that metabolic flux configurations in cancer cells are determined by the trade-off among four biological objectives (Fig. 1a), including (1) maximization of biomass synthesis, which is frequently considered as the only objective of cancer cells in previous studies [17, 19, 23, 33]; (2) maximization of ATP production, which is considered as the objective of non-malignant cells in some studies [17, 20]; (3) minimization of total abundance of metabolic enzymes, which is an analogue of the solvent capacity constraint (i.e. total abundance of intracellular proteins is limited by molecular crowding in the cytoplasm) [16, 34], and (4) minimization of total carbon uptake [35]. These four objectives reflect different metabolic demands of cancer cells, therefore covering both maximization of yield and minimization of cost. Combining them with the human genome-scale metabolic model Recon 1 [36] (Additional file 2: Table S1), we created a multi-objective optimization model (Fig. 1a, Additional file 1: Supplementary Methods), which lays the theoretical foundation for our subsequent analysis.

Based on this model, we quantitatively describe trade-offs among the four metabolic objectives by considering metabolic flux configurations with Pareto optimality. Pareto optimality is defined by the inability to simultaneously improve performances on all objectives. For instance, if a metabolic flux configuration has Pareto optimality with respect to the two objectives of maximizing biomass and maximizing ATP yield, there shall be no other flux configurations that yield both higher biomass synthesis and higher ATP production. To uniformly sample from all flux configurations with Pareto optimality (i.e. Pareto solutions), we designed an algorithm based on the ε-constraint method [37]. Briefly, this method transformed the original multi-objective optimization problem to a collection of single-objective linear programming problems (e.g. maximizing biomass synthesis subjected to constraints on ATP production, enzyme abundance and carbon uptake), which were then optimized to generate solutions with Pareto optimality with respect to the four objectives. Using this method, we have sampled 42,930 Pareto solutions in total (Additional file 1: Supplementary Methods). In line with potential objective trade-offs, these Pareto solutions exhibit substantial variabilities in each single metabolic objective and coupling between different objectives (Fig. 1b, c). In summary, these results confirm that the multi-objective optimization model can simulate a broad spectrum of metabolic flux configurations, yielding different amounts of energy and building blocks with variable engagements of metabolic enzymes and nutrients.

### Pareto models accurately predict metabolic phenotypes of cancer cells

**Fig. 2:**
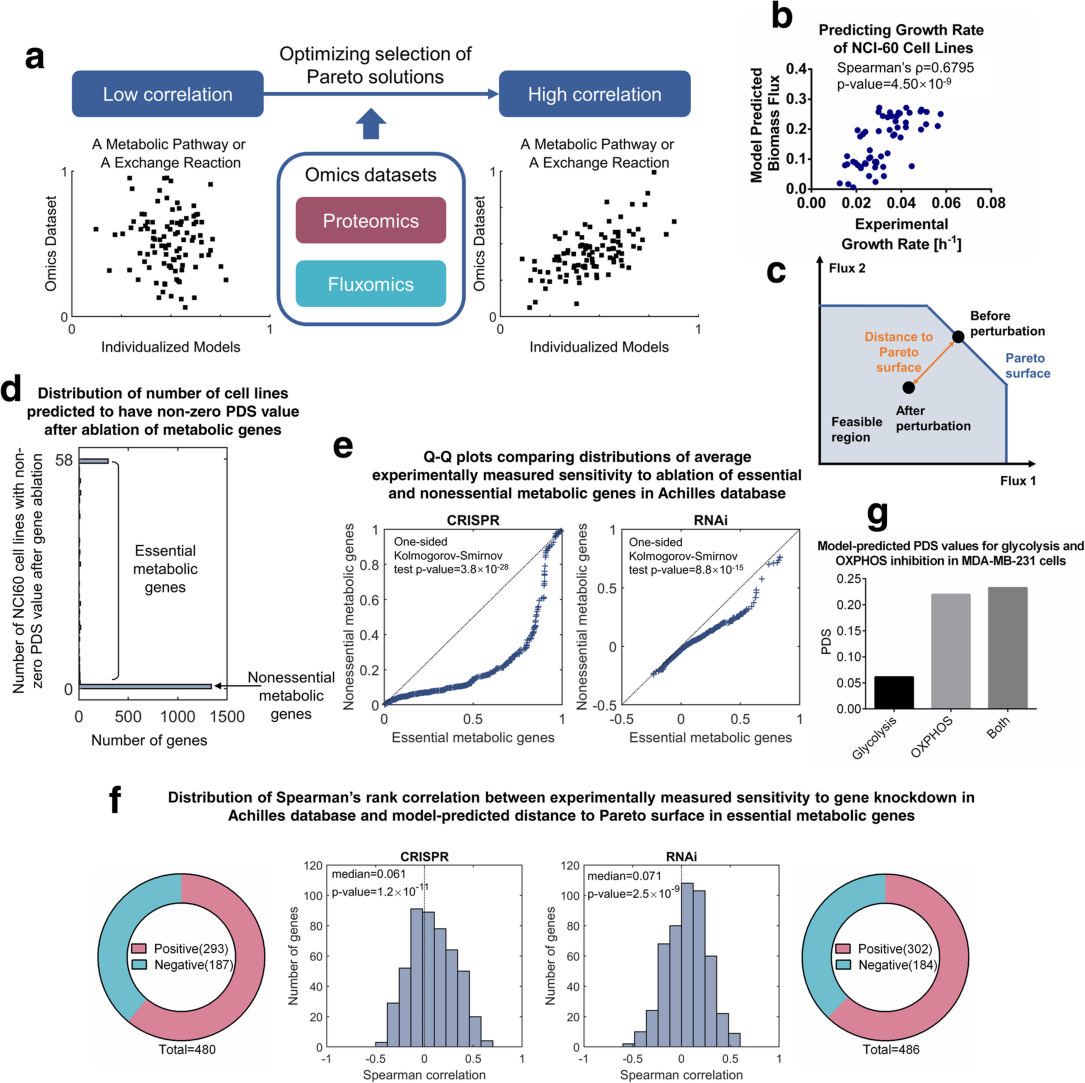
Pareto models accurately predict metabolic phenotypes of cancer cells. (**a**) Illustration of the strategy used in constructing the cell line-specific models based on multiple omics datasets. (**b**) Comparison between actual and model-predicted cell growth rates in the NCI-60 cancer cell panel. The *p*-value was computed using permutation test. (**c**) Illustration of Pareto deviation score (PDS) as a metric quantifying the impact of metabolic perturbation on cell viability. (**d**) Distribution of number of NCI-60 cell lines with non-zero PDS values after gene ablation in metabolic genes. (**e**) Quantile-quantile (Q-Q) plots comparing distributions of experimentally measured sensitivity to gene ablations between essential and nonessential metabolic genes. Left panel: CRISPR-based dataset; right panel: RNAi-based dataset. *P*-values were computed using one-sided Kolmogorov-Smirnov test. (**f**) Distributions of Spearman’s rank correlation coefficients between experimentally measured sensitivity to gene ablations and model-predicted PDS values in essential metabolic genes. *P*-values were computed using one-sided Wilcoxon’s signed rank test. Left panel: CRISPR-based dataset; right panel: RNAi-based dataset

To further validate the four-objective optimization method in modeling cancer metabolism, we next integrated the Pareto solutions with multi-omics datasets for a collection of cancer cell lines to construct cell line-specific models and validated these models with reported experimental data. Briefly, we constructed specific models by searching for a group of Pareto solutions maximizing the similarity between metabolic enzyme abundances or metabolite exchange fluxes and metabolic fluxes in the corresponding Pareto solutions (Fig. 2a). This was based on the assumption that for a particular metabolic pathway, the total abundance of metabolic enzymes associated with it closely correlates with the metabolic flux through this pathway. We used multi-omics datasets including LC-MS/MS based proteomics [38] and consumption-release (CORE) profiles of metabolites [39] to reconstruct Pareto models for NCI-60 cell lines (Supplementary Methods).

We first validated the Pareto models by comparing model-predicted biomass fluxes to the actual cell growth rates. The model-predicted biomass production fluxes significantly correlate with experimentally measured cell growth rates (Fig. 2b, Spearman’s rank correlation coefficient = 0.68, *p*-value = 4.5 × 10^− 9^), demonstrating that the cell line-specific Pareto models successfully recapitulate phenotypes of examined cancer cells. To validate that all four metabolic objectives are indispensable in predicting cell growth rates, we repeated this analysis with alternative models constructed with fewer metabolic objectives (Additional file 1: Fig. S1a-d) or less omics data input (proteomics only or exchange fluxes only, Additional file 1: Fig. S1e, f) and found that the prediction accuracy was significantly decreased in these cases. We also compared our Pareto models with models constructed using other computational approaches, including E-Flux, which directly uses expression levels of metabolic genes to adjust upper limits of associated fluxes [40], and personalized reconstruction of metabolic models (PRIME), which utilizes prior knowledge about correlations between metabolic gene expressions and cellular phenotypes to improve prediction accuracy [19]. Using our input dataset, none of these approaches was able to predict cell growth rates as well as our model did (Additional file 1: Fig. S2a-d).

Next, we applied this model to predict cellular responses to metabolic gene ablations and compared the calculated results with experimentally measured, cell line specific sensitivities to RNAi-mediated [41] and CRISPR/Cas9-mediated [42] gene ablations in Achilles, a genome-scale gene essentiality database [43]. Based on the assumption that cancer cells tend to adopt flux configurations with Pareto optimality, we hypothesize that the deviation of a metabolic flux configuration from the Pareto surface reflects the fitness of cells bearing such a flux configuration. To quantify this deviation, we computed a Pareto deviation score (PDS) defined as the Euclidean distance between the flux configuration and the Pareto surface (Fig. 2c, Additional file 1: Supplementary Methods). It is thus straightforward that all PDS values are non-negative, and a metabolic flux configuration has a PDS value equal to zero only if it is a Pareto solution. For each metabolic gene (1905 in total) included in the model, we evaluated its essentiality in NCI-60 cell lines by computing the PDS values for the flux configurations after genetic knockdown. We found that most (1341 out of 1905) metabolic genes were associated with zero PDS values in all NCI-60 cell lines, suggesting that perturbation of these genes has minimal influence on fitness of the cells. We thus defined these genes as nonessential metabolic genes and genes with non-zero PDS values in at least one cell line as essential metabolic genes (Fig. 2d). Compared to nonessential metabolic genes, essential genes predicted by the model were associated with reactions carrying higher fluxes (Additional file 1: Fig. S3b) and pathways known to be important in energy and biomass production (Additional file 1: Fig. S3c,d). Cell lines in the Achilles database were also in general more sensitive to ablations of model-predicted essential metabolic genes (Fig. 2e). These results together demonstrate that PDS correctly predict metabolic genes essential for survival in different cancer cell lines.

We next examined whether PDS also correctly predicted cell line specific response to metabolic perturbations. We correlated model-predicted PDS values with experimentally measured sensitivities to CRISPR/Cas9-mediated and RNAi-mediated gene ablations in the Achilles database and found that, for most of the examined genes (293 out of 480 in the CRISPR/Cas9-based dataset and 302 out of 486 in the RNAi-based dataset), the PDS values positively correlated with experimentally-determined sensitivity scores in cell lines shared by the NCI-60 panel and the Achilles database (Fig. 2f, median Spearman correlation = 0.061 for the CRISPR/Cas9-based dataset and 0.071 for the RNAi-based dataset, one-sided Wilcoxon’s signed rank *p*-value = 1.2 × 10^− 11^ for the CRISPR/Cas9-based dataset and 2.5 × 10^− 9^ for the RNAi-based dataset), suggesting that model-predicted deviations from the Pareto surface correctly predict cell line-specific sensitivities to metabolic gene ablations. We also tested several other metrics including model-predicted reduction in growth rates, mRNA expression levels and protein abundance. We compared them with the PDS values in terms of their abilities to predict cellular responses to metabolic gene ablation and found that the PDS metric yielded the best results (Additional file 1: Fig. S4). Moreover, model-predicted PDS values were also consistent with a recent finding that combinatorial inhibition of glycolysis and OXPHOS resulted was necessary to eliminate MDA-MB-231 cancer cells [44], in which the combinatorial treatment resulted in a larger PDS value compared to inhibiting either glycolysis or OXPHOS (Fig. 2g). These results demonstrate that the Pareto models predict cellular phenotypes more accurately than other computational approaches examined and suggest that impairment of Pareto optimality by perturbing expressions of metabolic enzymes correlates with fitness reduction in cancer cells. It is thus promising to apply this approach in designing potential therapeutics that selectively target cancer cells with aggressive metabolic phenotypes.

### Metabolic targets identified by Pareto surface analysis are essential for cancer progression

**Fig. 3:**
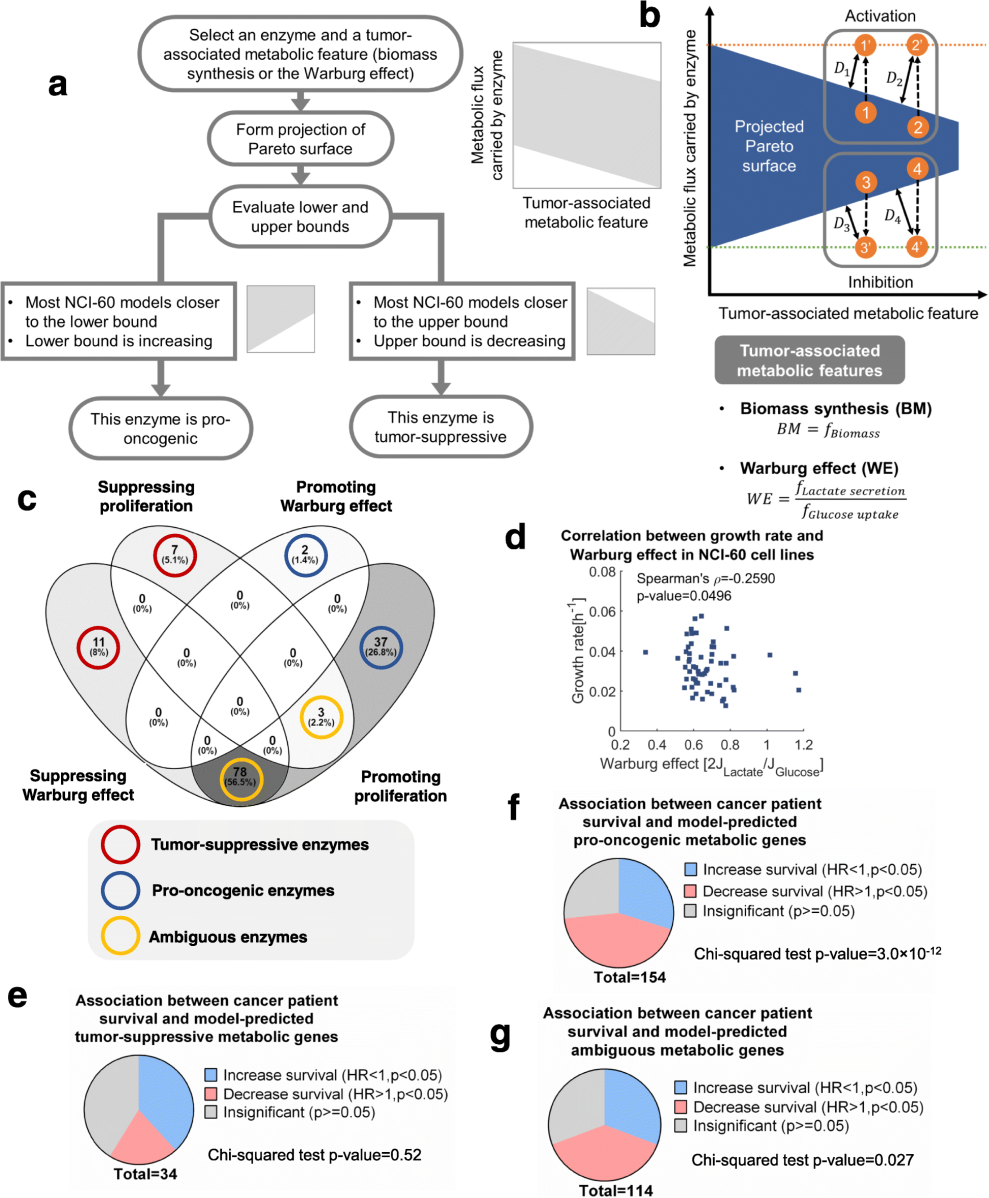
Metabolic targets identified by Pareto surface analysis correlate with cancer progression and patient prognosis. (**a**) Workflow of identifying potential metabolic targets essential for cell proliferation and the Warburg effect. (**b**) Illustration of the criteria for target identification. (**c**) Venn diagram showing the overlap between model-predicted proliferation-suppressing, proliferation-promoting, Warburg effect-suppressing and Warburg effect-promoting enzymes. (**d**) Correlation between growth rate and the Warburg effect in NCI-60 cell lines. The p-value was computed using permutation test. (**e**) Fraction of genes with different relationships to breast cancer patient survival in model-predicted tumor-suppressive metabolic genes. (**f**) Same as in (**e**) but for model-predicted pro-oncogenic metabolic genes. (**g**) Same as in (**e**) cmetabolic genes

Given that model-predicted impairment of Pareto optimality reflects sensitivities to metabolic perturbation, we next sought to identify anti-tumor metabolic targets using this approach. The goal was to identify metabolic genes and enzymes whose activation or inhibition lead to selective impairment of viabilities in cells with cancer-associated metabolic features such as rapid proliferation and the Warburg effect. Therefore, we designed a perturbation strategy leading to larger Pareto deviation in flux configurations with higher biomass production or stronger Warburg effect, aiming to selectively reduce the viability of malignant cells (Fig. 3a). This strategy was achieved by activation or inhibition of metabolic enzymes, which can be quantified in our model as increased or decreased metabolic fluxes governed by this particular enzyme. Without loss of generality, we use cell growth rate as a representative phenotype to illustrate our strategy for target identification. First, we projected the Pareto surface to a two-dimensional space spanned by growth rate and one specific metabolic flux, in a way that we can clearly define its lower and upper bounds (Fig. 3a). After that, we examined how the upper bound of metabolic flux varies with cell growth rate. If the upper bound decreases with growth rate, activation of this enzyme would lead to larger impairment of Pareto optimality for flux configurations with higher growth rate, thus resulting in selective fitness impairment towards highly proliferative cells. In this case the enzyme is considered to be tumor-suppressive (Fig. 3b). Conversely, inhibition of an enzyme would selectively impair the viability of fast-growing cancer cells, if the lower bound rises monotonously with their growth rates. This enzyme is thus considered to be pro-oncogenic (Fig. 3b). A correlation-based monotonousness score was defined to assess the tendency of declining upper bound or rising lower bound (Supplementary Methods, Additional file 3: Table S2). We also require that most of the individualized models for NCI-60 cell lines locate close to the upper or lower boundary, to allow the metabolic perturbation to draw the flux configurations out of the Pareto surface and confer significant impact on cell viability.

By analyzing the geometry of projected Pareto surface as introduced above, we identified four groups of metabolic enzymes that either suppress or promote cancer cell growth or the Warburg effect (Fig. 3c, Additional file 4: Table S3). Notably, these four categories of enzymes had little overlap with each other except between enzymes promoting proliferation and those suppressing the Warburg effect (78 overlapped enzymes out of 89 Warburg effect-suppressing enzymes and 115 proliferation-promoting enzymes). No versatile target was predicted to exist whose activation or inhibition is able to inhibit both processes. This result seems to be contradictory to several previous studies [45, 46]. However, it is worth mentioning that our modeling results only reflect the direct consequence of metabolic perturbation. Metabolic enzymes often carry essential non-metabolic functions, and inhibition of cell proliferation may lead to metabolic shifts secondary to growth arrest, which were not considered by our analysis or most other theoretical methods. Nevertheless, we also found a significant negative correlation between growth rate and the Warburg effect in NCI-60 cell lines (Fig. 3d, Spearman’s correlation = − 0.2590, *p*-value = 0.0496), suggesting a plausible trade-off between proliferation and the Warburg effect. The finding about the contradictory roles of the ambiguous enzymes in promoting cell proliferation and suppressing the Warburg effect was also supported by several lines of literature-based evidence. For instance, the model-predicted ambiguous enzyme, acetyl-CoA carboxylase (ACC), was shown to shift cancer metabolism from glycolysis-dependent to lipogenesis-dependent in human head and neck squamous cell carcinoma (HNSCC) cells [47] and suppress whole-body glycolysis in high-fat-fed mice [48], while its inhibition impaired proliferation of human prostate cancer cells [49]. Another model-predicted ambiguous enzyme, proline dehydrogenase (PRODH/POX), was shown to suppress lactate production in human colon cancer cells [50], while its inhibition impaired proliferation of human lung cancer cells [51] and breast cancer cells [52]. Besides the 89 ambiguous enzymes, there are 18 enzymes predicted to inhibit either proliferation or the Warburg effect, and 39 enzymes predicted to function in the opposite direction (Additional file 5: Table S4). We reasoned that the latter two groups of enzymes may serve as a potential target pool for therapeutic intervention, especially those harboring expression profiles significantly correlated with disease progression.

To validate the association between identified metabolic targets and cancer progression, we conducted Kaplan-Meier survival analysis on thousands of breast cancer patients to systematically evaluate the connection between metabolic enzymes and patient survival [53]. Specifically, we analyzed the correlation between gene expression levels and relapse-free survival of breast cancer patients for all metabolic genes, and divided these genes to three categories whose up-regulation positively, negatively or non-significantly correlates with patient prognosis (Additional file 6: Table S5). Consistent with model predictions, expression levels of tumor-suppressive metabolic genes were more likely to associate with better patient survival (Fig. 3e), and model-predicted pro-oncogenic genes generally associate with worse prognosis (Chi-squared test *p*-value = 3 × 10^− 12^, Fig. 3f). For the ambiguous gene set, the trends of survival correlations were lying in between the cases of pro-oncogenic and tumor-suppressive ones (Fig. 3g). Taken together, these integrated analyses of omics datasets validated the close association between model-predicted targets and cancer progression. Altering the cutoff parameters used in the Pareto surface analysis had little effects on the correlation between model-predicted putative targets and patient survival (Additional file 1: Fig. S5), suggesting that the Pareto surface analysis approach is robust to parameter selections. Notably, targets identified by the Pareto surface analysis had little overlap with and showed better concordance with patient survival than targets identified from previously-applied metrics, including reduction of growth rate simulated using minimization of metabolic adjustment (MOMA) [32], correlation between cancer cell proliferation and mRNA expression, and correlation between cancer cell proliferation and protein abundance (Additional file 1: Fig. S6, Additional file 1: Supplementary Methods), suggesting that our analysis may identify more novel targets potentially exploited for cancer therapeutics.

### Experimental validation of identified metabolic targets

Based on above analyses, we have identified several groups of metabolic enzymes whose up- or down-regulation is potentially essential for cancer progression. These enzymes may serve as novel targets for designing anti-tumor therapeutics. We next sought to validate these targets in cell-based experiments. Since only two metabolic enzymes, lactate dehydrogenase (LDH) and monocarboxylate transporter (MCT), were predicted to promote the Warburg effect (Fig. 3c) and consistent with their well-established functions, we decided to validate metabolic targets falling in the other three categories, i.e. proliferation-promoting enzymes, proliferation-suppressing enzymes, and Warburg effect-suppressing enzymes.

**Fig. 4:**
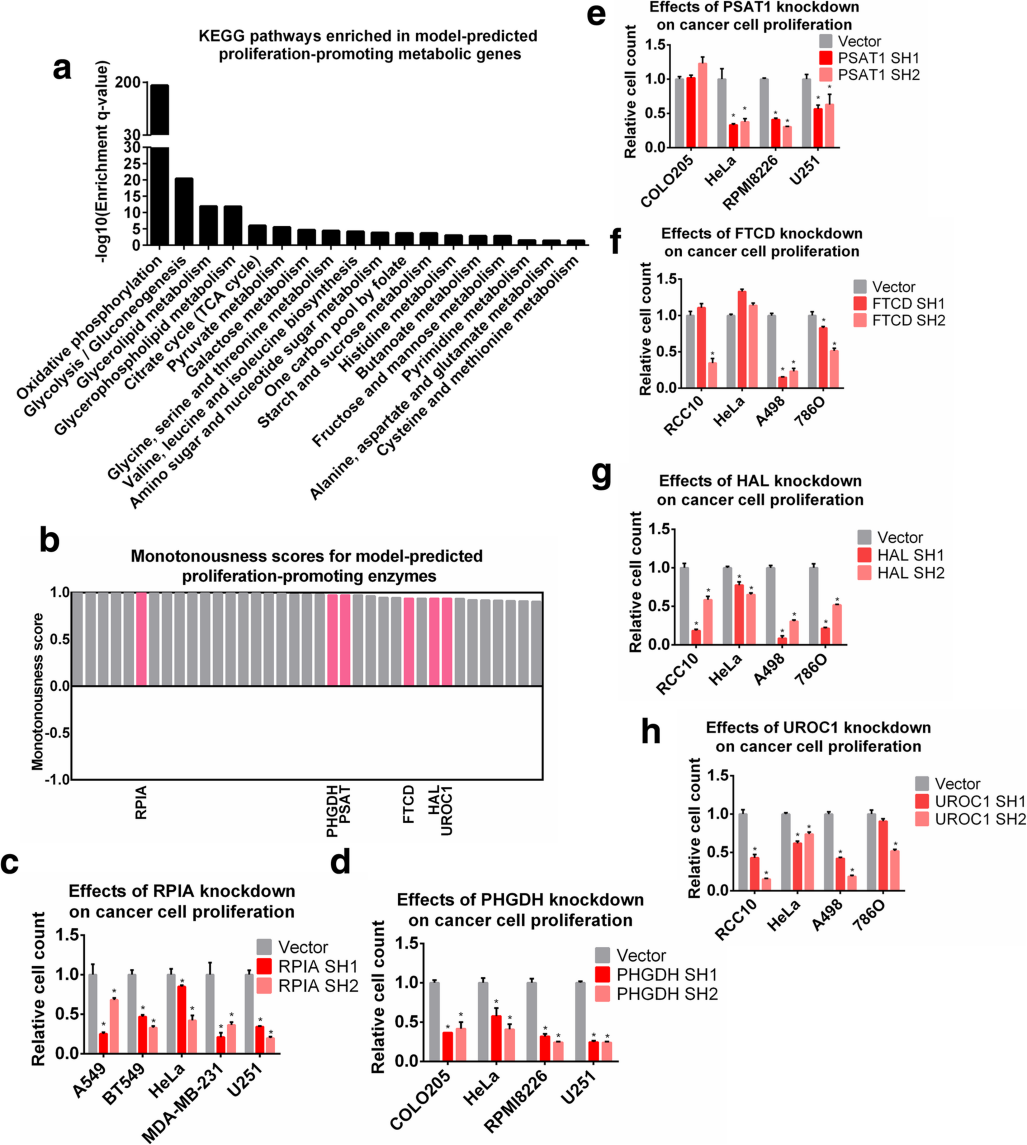
Experimental validation of proliferation-promoting targets. (**a**) KEGG pathways enriched in model-predicted proliferation-promoting targets. (**b**) Monotonousness scores for model-predicted proliferation-promoting enzymes. Enzymes selected for experimental validation are highlighted in red. (**c**-**h**) Relative number of cells after 4 days upon shRNA knockdown of (**c**) RPIA; (d) PHGDH; (**e**) PSAT1; (**f**) FTCD; (g) HAL; (**h**) UROC1 in the tested cell lines. *P*-values were computed using Wilcoxon’s rank sum test. P-value< 0.05 was considered as significant

We first examined the group of proliferation-promoting metabolic enzymes. Pathway enrichment analysis revealed that these enzymes were enriched in many metabolic pathways known to be up-regulated in tumors, such as glycolysis, TCA cycle, oxidative phosphorylation, nucleotide metabolism and serine, glycine and one carbon metabolism (Fig. 4a), thus supporting the effectiveness of the Pareto approach in identifying anti-tumor metabolic targets. Moreover, among this class of targets, there are also some novel candidates whose functions in cancer have not been thoroughly investigated, including three enzymes in the histidine degradation pathway, formimidoyltransferase cyclodeaminase (FTCD), histidase (HAL), and urocanase (UROC). We chose these enzymes for experimental validation, as well as three other enzymes that have been investigated in recent cancer studies, namely ribose-5-phosphate isomerase (RPI), phosphoglycerate dehydrogenase (PHGDH) and phosphoserine transaminase (PSAT) as positive controls. All these enzymes were highly scored in the Pareto surface analysis (Fig. 4b). Most cell lines used in our experiments were selected from the NCI-60 panel, whereas some unavailable lines were replaced by alternatives with identical cancer types. HeLa was also included as a control. Efficiencies of gene ablations were validated by RT-PCR (Additional file 1: Fig. S7). Knockdown of each individual target was associated with strong anti-proliferative effects in examined cell lines (Fig. 4c-h). These results demonstrated that the model-predicted proliferation-promoting enzymes, including those involved in histidine degradation, are indeed essential for cancer cell proliferation.

### Activation of lysine degradation pathway impairs cancer cell proliferation

**Fig. 5:**
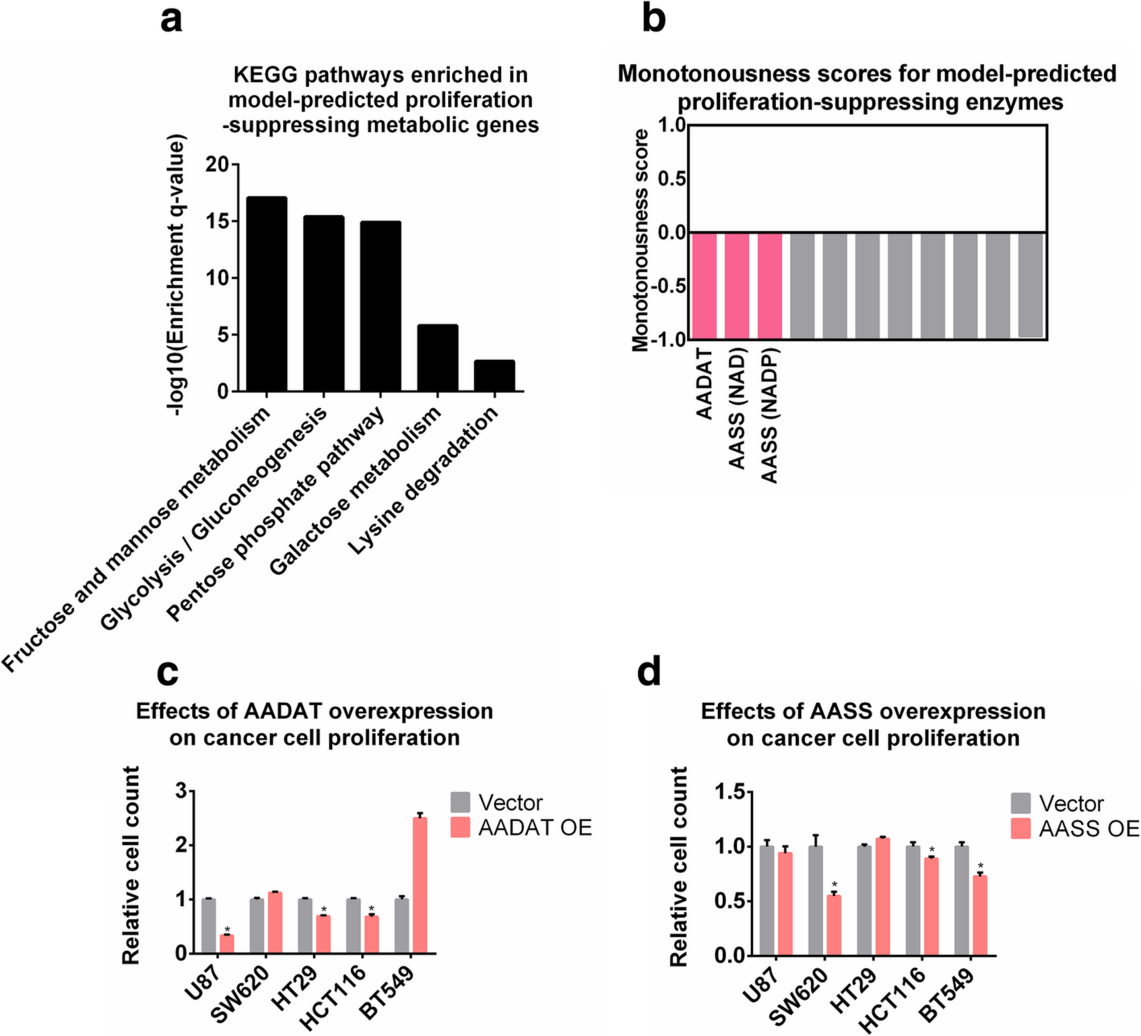
Activation of lysine degradation pathway impairs cancer cell proliferation. (**a**) KEGG pathways enriched in model-predicted proliferation-suppressing targets. (**b**) Monotonousness scores for model-predicted proliferation-suppressing enzymes. Enzymes selected for experimental validation are highlighted in red. (**c**) Relative numbers of cells after 4 days upon AADAT over-expression in the tested cell lines. OE: over-expression. *P*-values were computed using Wilcoxon’s rank sum test. *P*-value< 0.05 was considered as significant. (**d**) Relative numbers of cells after 4 days upon AASS over-expression in the tested cell lines. *P*-values were computed using Wilcoxon’s rank sum test. *P*-value< 0.05 was considered as significant

Besides the putative proliferation-promoting metabolic enzymes we have identified and validated, this study also highlighted several novel pathways functioning in the opposite direction. In particular, our model predicted 7 metabolic enzymes to be potentially proliferation-suppressing, indicating that up-regulation of these enzymes may help restrain tumor growth. These enzymes are enriched within metabolic pathways related to carbohydrate anabolism and lysine degradation (Fig. 5a). Among them, the top-scored three are aminoadipate-semialdehyde dehydrogenase (NAD- or NADP-dependent) and 2-aminoadipate transaminase (Fig. 5b). These enzymes catalyze the first few steps (1st, 2nd and 4th steps) of lysine degradation pathway, thus potentially controlling the metabolic flux through this route. To validate the prediction that activating these enzymes may help reduce cancer cell growth, we over-expressed two related genes, AASS and AADAT, in a series of cancer cell lines with different tissues-of-origin and genetic backgrounds. Efficiencies of over-expressions were confirmed by Western blot analyses (Additional file 1: Fig. S7). In line with the predictions of our computational model, over-expression of AADAT or AASS significantly inhibited proliferation in 3 out of the 5 tested cell lines (Fig. 5c, d), corroborating the model-predicted rationality of suppressing cancer cell proliferation via activation of the lysine degradation pathway.

### Over-expression of selective metabolic enzymes inhibits the Warburg effect

**Fig. 6:**
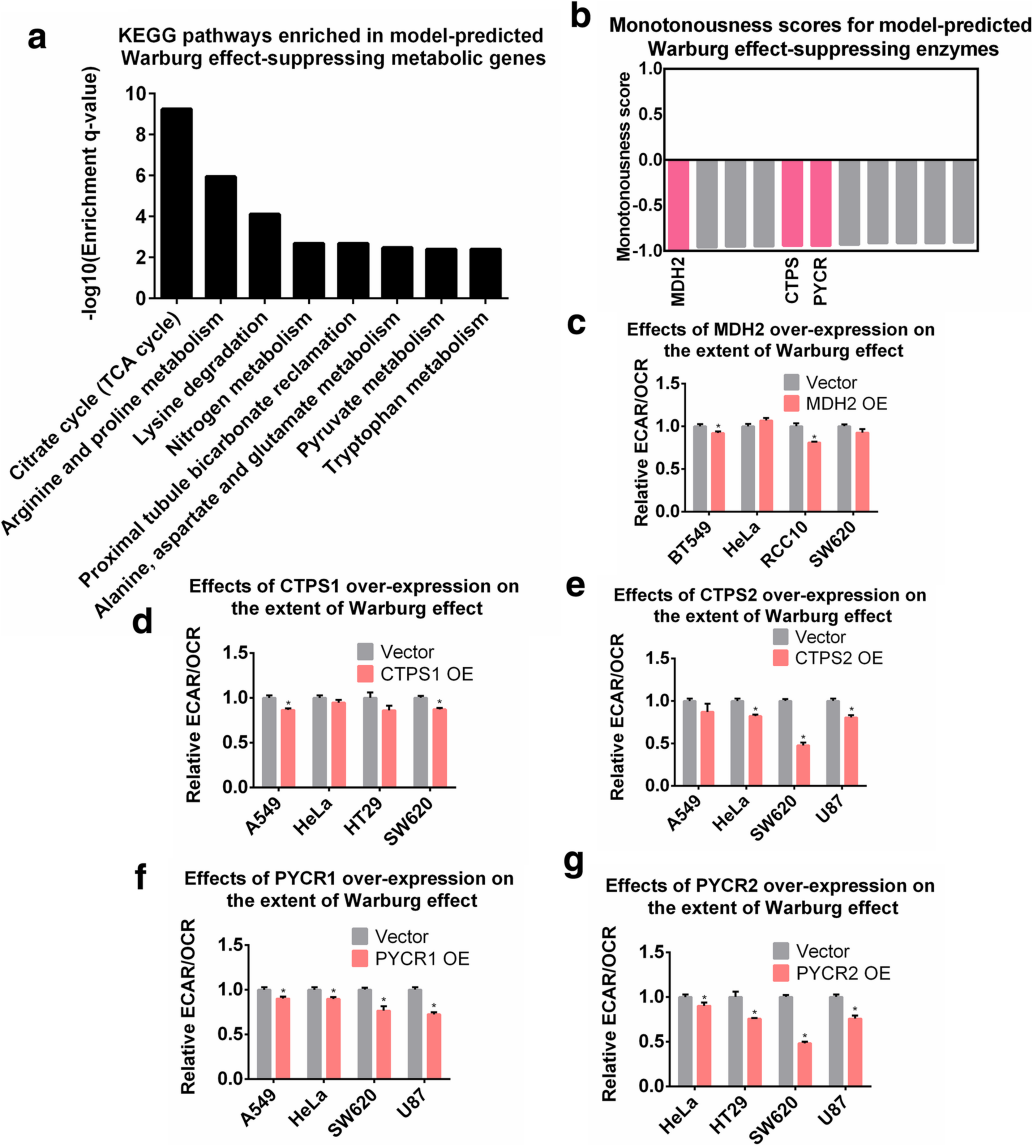
Over-expression of metabolic enzymes inhibits the Warburg effect. (**a**) KEGG pathways enriched in model-predicted Warburg effect-suppressing targets. (**b**) Monotonousness scores for model-predicted Warburg effect-suppressing enzymes. Enzymes selected for experimental validation are highlighted in red. (**c**-**g**) Relative values of ECAR/OCR ratio after 4 days upon (**c**) MDH2; (**d**) CTPS1; (**e**) CTPS2; (**f**) PYCR1; (**g**) PYCR2 over-expression in the tested cell lines. P-values were computed using Wilcoxon’s rank sum test. P-value< 0.05 was considered as significant

Another interesting prediction by our Pareto model is the wide existence of putative Warburg effect-suppressing metabolic enzymes. Pathway enrichment analysis showed that these enzymes were related to the TCA cycle, amino acid metabolism and several other functional categories (Fig. 6a). Many of them can direct metabolic fluxes away from lactate production, thereby inhibiting the Warburg effect. We selected three of the top-scored enzymes including mitochondrial malate dehydrogenase (MDH2) in the TCA cycle, CTP synthase (CTPS) in pyrimidine metabolism, and pyrroline-5-carboxylate reductase (PYCR) in arginine and proline metabolism for experimental validation (Fig. 6b). We quantitated the magnitude of the Warburg effect by the ratio of extracellular acidification rate (ECAR) to oxygen consumption rate (OCR) (Additional file 1: Fig. S8 and Supplementary Methods) in 7 cell lines after the individual over-expression of MDH2 (Fig. 6c), CTPS1 (Fig. 6d), CTPS2 (Fig. 6e), PYCR1 (Fig. 6f) or PYCR2 (Fig. 6g). Indeed, we found that over-expression of these metabolic genes, especially PYCR1, PYCR2 and CTPS2, resulted in a great inhibition of the Warburg effect in multiple cell lines. These metabolic enzymes have not been extensively characterized in the field of cancer metabolism, thus representing interesting targets for future investigation. Moreover, up-regulation of these metabolic genes did not increase cell growth rate except for the SW620 cell line (Additional file 1: Fig. S9), which is consistent with our predictions that these enzymes did not serve as putative proliferation-promoting factors.

In summary, using the multi-objective optimization model of cancer metabolism and the Pareto surface analysis strategy, we have identified an array of metabolic enzymes that significantly regulate cancer-associated phenotypes including cell proliferation and the Warburg effect. We have validated predicted results using cell-based assays, demonstrating that these enzymes may serve as metabolic targets for exploiting novel cancer therapeutics.

## Discussion

In this study, we developed a novel strategy to model cancer metabolism based on the assumption of multi-objective optimization. Specifically, we applied the concept of Pareto optimality to predict metabolic flux configurations with optimality in balancing the demands for maximization of yields (growth and energy) and minimization of costs (enzymes and nutrients). By integrating these metabolic objectives with multi-omics datasets, we were able to construct cell line-specific models that correctly predicted multiple phenotypes of cancer cells including cell growth rates and responses to metabolic perturbations. This is the first attempt, to our best knowledge, to incorporate multiple objectives in modeling cancer metabolism, which demonstrates that theoretically estimated deviations from Pareto optimality with respect to different metabolic goals closely resemble impairments of cell viability.

In our current model, we selected 4 most commonly utilized metabolic objectives for FBA analysis, including maximization of biomass production, maximization of ATP hydrolysis, minimization of total abundance of metabolic enzymes, and minimization of total carbon uptake. Nevertheless, some other objectives may also be considered, such as minimization of redox imbalance, maximization of resistance to cytotoxic agents, minimization of reactive oxygen species (ROS) production, etc. Incorporating additional objectives in our model may further improve the fitting accuracy of Pareto surfaces to the actual metabolic configurations of cells and tissues under different circumstances. Strategies to deduct the best combinations of objectives [54, 55] may be combined with our modeling method, and provide new insights to the reprogramming mechanism of cancer metabolism.

Our theoretical model successfully dissects the cancer metabolic network and identifies its vulnerabilities from a global perspective. More specifically, we determined several groups of novel metabolic targets controlling cancer cell proliferation or the Warburg effect by analyzing the geometry of the Pareto surface. Some of these targets were not identified by previously reported approaches, suggesting that the assumption of multiple metabolic goals is essential for identification of these novel targets in the metabolic network. The model-predicted associations between these targets and cancer progression were not only consistent with their correlations with patient survival, but also experimentally validated in a series of cancer cell lines with different backgrounds. Investigation of these target categories revealed several metabolic pathways with important yet understudied functions in cancer progression, such as histidine and lysine metabolism. Activity of histidine degradation pathway has recently been shown to positively correlate with sensitivity to methotrexate [56], suggesting that activating this pathway could be beneficial by enhancing effectiveness of related chemotherapies. However, our study indicates that this pathway can also be pro-oncogenic by promoting cancer cell growth. The exact roles of these pathways in different cancer types need further investigation. Taken together, our theoretical and experimental results suggest that novel roles of metabolic enzymes in cancer progression can be uncovered by analyzing the landscape of Pareto surface under the framework of four-objective optimization.

Moreover, our model highlighted a contradictory role played by several metabolic enzymes in affecting cell growth and the Warburg effect. For a group of enzymes identified as potential targets for rapid proliferation, their activations were predicted to inhibit the Warburg effect (ambiguous enzymes in Fig. 3c). The conflict between inhibiting cell proliferation and the Warburg effect reflects the intrinsic robustness of cancer as a complex disease. However, this could also be due to the fact that our modeling approach only considers the direct influence of metabolic perturbation, not the secondary effects derived from primary manipulations. In addition, our method only incorporated the stoichiometric constraints of metabolic fluxes, and ignored nonlinear factors such as the allosteric regulation of metabolic enzymes for modeling feasibilities. Further experimental investigation is needed to characterize the precise roles of those enzymes in cancer. Nevertheless, we presented a comprehensive strategy to identify cancer-associated vulnerabilities with much-improved accuracies, as supported by our survival analyses and cell-based experiments.

## Conclusions

To summarize, we have developed a novel method to model cancer metabolism based on Pareto optimality under the framework of multi-objective optimization. This approach created an integrated workflow from omics-based mathematical models to metabolic target identification, and predicted metabolic hubs essential for cancer cell proliferation and/or the Warburg effect. The high concordance between predicted roles of metabolic enzymes in cancer and tumor ‘omics’ data suggests that the overall effect of a specific enzyme during tumor development should be determined by its comprehensive functions in multiple cellular tasks, rather than a single task such as cell proliferation. In addition to modeling cancer metabolism, this methodology may also be applied to explore other disease-related metabolic abnormalities with accessible omics datasets.

## Supplementary information

**Additional file 1: **Supplementary materials and methods. **Fig. S1.** (Related to Fig. 2). Comparison between actual growth rates of NCI-60 cell lines and growth rates predicted by alternative models. **Fig. S2** (Related to Fig. 2). Comparisons between actual growth rates of NCI-60 cell lines and growth rates predicted by other methods. **Fig. S3.** (Related to Fig. 2). Comparison between model-predicted essential and nonessential metabolic genes. **Fig. S4.** (Related to Fig. 2). Distributions of Spearman’s rank correlation coefficients between experimentally measured sensitivities of NCI-60 cell lines to metabolic gene knockdowns and sensitivities predicted by different computational methods. **Fig. S5**. (Related to Fig. 3). Influences of monotonousness score cutoff used in Pareto surface analysis on the associations between model-predicted targets and cancer patient survival. **Fig. S6.** (Related to Fig. 3). Comparison between targets identified by Pareto surface analysis and other methods. **Fig. S7.** (Related to Figs. 4, 5, 6). Validation of efficiencies of gene knockdowns and over-expressions. **Fig. S8.** (Related to Fig. 6). Mitochondrial respiration and ECAR profiles of SW620, A549, BT549, HeLa, RCC10 and U87 cells with or without over-expression of MDH2, CTPS1, CTPS2, PYCR1 or PYCR2. **Fig. S9.** (Related to Fig. 6). Relative number of cells after 4 days in the control group (PCDH) or upon over-expression of MDH2, CTPS1, CTPS2, PYRC1 or PYRC2 in the tested cell lines. (DOCX 4356 kb)

**Additional file 2: Table S1.** Information of the genome-scale metabolic model used in this study. (XLSX 732 kb)

**Additional file 3: Table S2.** Monotonousness scores for all metabolic enzymes included in the model. (XLSX 127 kb)

**Additional file 4: Table S3.** Lists of metabolic targets identified based on the Pareto surface analysis. (XLSX 20 kb)

**Additional file 5: Table S4.** Complete lists of tumor-suppressive, pro-oncogenic and ambiguous enzymes and genes. (XLSX 25 kb)

**Additional file 6: Table S5.** Complete results of survival analysis for all metabolic genes included in the model. (XLSX 59 kb)

## Data Availability

The datasets generated in this study are available in the figshare repository: https://figshare.com/articles/Multi-objective_optimization_model_of_cancer_metabolism/8182331. The omics datasets analyzed in this study are available in repositories detailed in the section “Retrieving and processing the omics datasets” in Supplementary Methods.

## Abbreviations

ACC: Acetyl-CoA carboxylase
CORE: Consumption-release
CTPS: CTP synthase
ECAR: Extracellular acidification rate
FBA: Flux balance analysis
FTCD: Formimidoyltransferase cyclodeaminase
GSMM: Genome-scale metabolic model
HAL: Histidase
HNSCC: Head and neck squamous cell carcinoma
LDH: Lactate dehydrogenase
MCT: Monocarboxylate transporter
MDH2: Mitochondrial malate dehydrogenase
MOMA: Minimization of metabolic adjustments
OCR: Oxygen consumption rate
PDS: Pareto deviation score
PHGDH: Phosphoglycerate dehydrogenase
PRIME: Personalized reconstruction of metabolic models
PRODH/POX: Proline dehydrogenase
PSAT: Phosphoserine transaminase
PYCR: Pyrroline-5-carboxylate reductase
RPI: Ribose-5-phosphate isomerase
UROC: Urocanase
