# Supplementary material for: Identification of Cancer–associated metabolic vulnerabilities by modeling multi-objective optimality in metabolism
Source: Cell Commun Signal. 2019 Oct 10;17:124. doi: 10.1186/s12964-019-0439-y (PMC6785927; doi:10.1186/s12964-019-0439-y)
Supplement: Supplementary file 1 — Additional file 1: Supplementary materials and methods. Fig. S1. (Related to Fig. 2). Comparison between actual growth rates of NCI-60 cell lines and growth rates predicted by alternative models. Fig. S2 (Related to Fig. 2). Comparisons between actual growth rates of NCI-60 cell lines and growth rates predicted by other methods. Fig. S3. (Related to Fig. 2). Comparison between model-predicted essential and nonessential metabolic genes. Fig. S4. (Related to Fig. 2). Distributions of Spearman’s rank correlation coefficients between experimentally measured sensitivities of NCI-60 cell lines to metabolic gene knockdowns and sensitivities predicted by different computational methods. Fig. S5. (Related to Fig. 3). Influences of monotonousness score cutoff used in Pareto surface analysis on the associations between model-predicted targets and cancer patient survival. Fig. S6. (Related to Fig. 3). Comparison between targets identified by Pareto surface analysis and other methods. Fig. S7. (Related to Figs. 4, 5, 6). Validation of efficiencies of gene knockdowns and over-expressions. Fig. S8. (Related to Fig. 6). Mitochondrial respiration and ECAR profiles of SW620, A549, BT549, HeLa, RCC10 and U87 cells with or without over-expression of MDH2, CTPS1, CTPS2, PYCR1 or PYCR2. Fig. S9. (Related to Fig. 6). Relative number of cells after 4 days in the control group (PCDH) or upon over-expression of MDH2, CTPS1, CTPS2, PYRC1 or PYRC2 in the tested cell lines. (DOCX 4356 kb) [file 12964_2019_439_MOESM1_ESM.docx]

**Additional files for**

**Identification of Cancer–associated Metabolic Vulnerabilities by Modeling Multi-objective Optimality in Metabolism**

Ziwei Dai^1^, Shiyu Yang^4^, Liyan Xu^4^, Hongrong Hu^4^, Kun Liao^4^, Jianghuang Wang^4^, Qian Wang^2^, Shuaishi Gao^1^, Bo Li^4*^, Luhua Lai^1,2,3*^

1. Center for Quantitative Biology, Academy for Advanced Interdisciplinary Studies, Peking University, Beijing 100871, China

2. Beijing National Laboratory for Molecular Sciences, State Key Laboratory for Structural Chemistry of Unstable and Stable Species, College of Chemistry and Molecular Engineering, Peking University, Beijing 100871, China

3. Peking-Tsinghua Center for Life Sciences, Peking University, Beijing 100871, China

4. Program of Cancer Research, Affiliated Guangzhou Women and Children's Hospital, Zhongshan School of Medicine, Sun Yat-Sen University, Guangzhou 510080, China


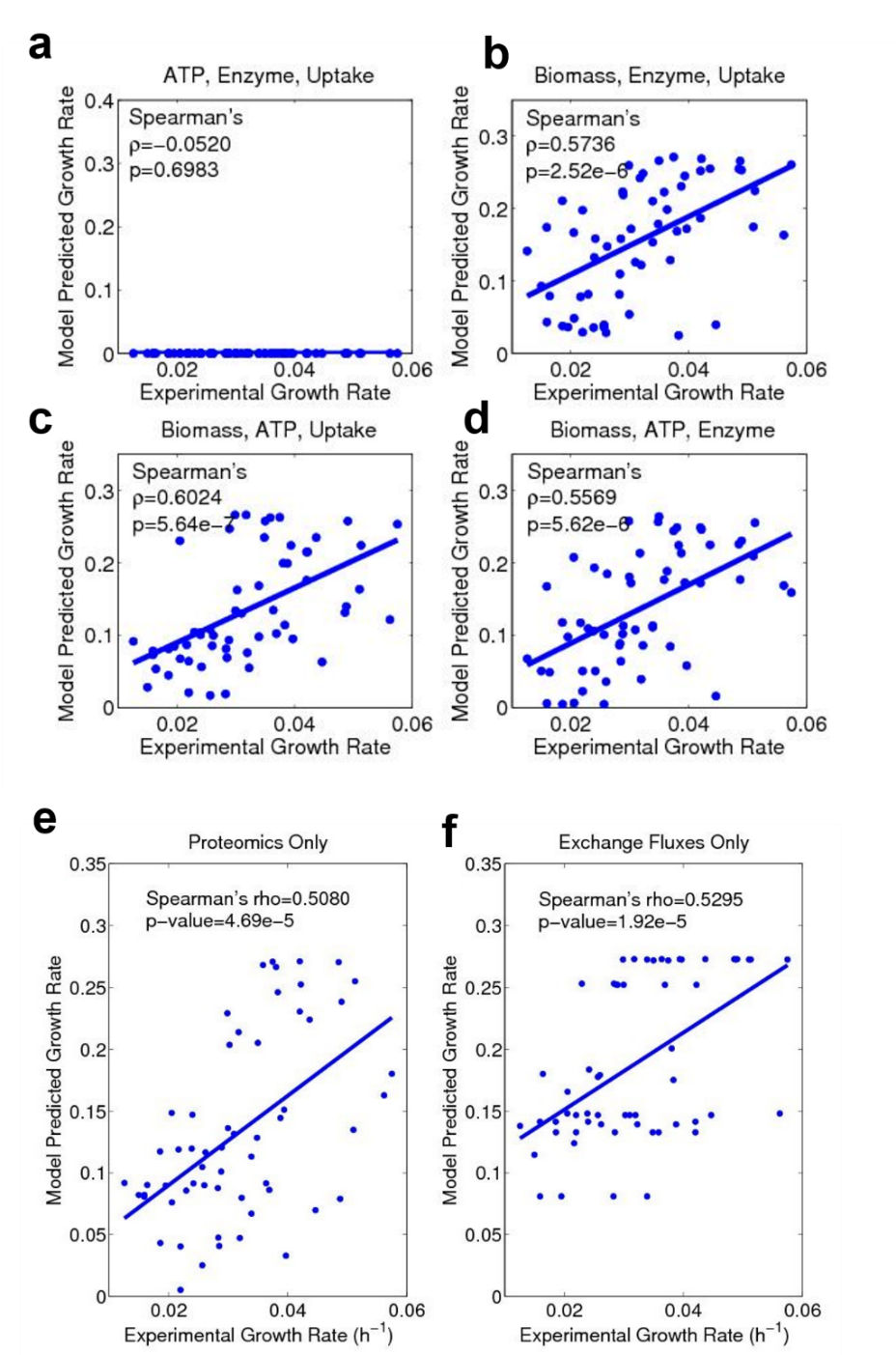


**Figure S1 (Related to Figure 2). Comparison between actual growth rates of NCI-60 cell lines and growth rates predicted by alternative models.**

(a-d) Comparisons between actual growth rates and growth rates predicted by multi-objective optimization models with three objectives: (a) maximizing ATP hydrolysis, minimizing enzyme abundance, minimizing carbon uptake; (b) maximizing biomass synthesis, minimizing enzyme abundance, minimizing carbon uptake; (c) maximizing biomass synthesis, maximizing ATP hydrolysis, minimizing carbon uptake; (d) maximizing biomass synthesis, maximizing ATP hydrolysis, minimizing enzyme abundance.

(e) Comparison between actual growth rates and growth rates predicted by cell line-specific models constructed using proteomics data as the only input dataset.

(f) Comparison between actual growth rates and growth rates predicted by cell line-specific models constructed using exchange flux data as the only input dataset.


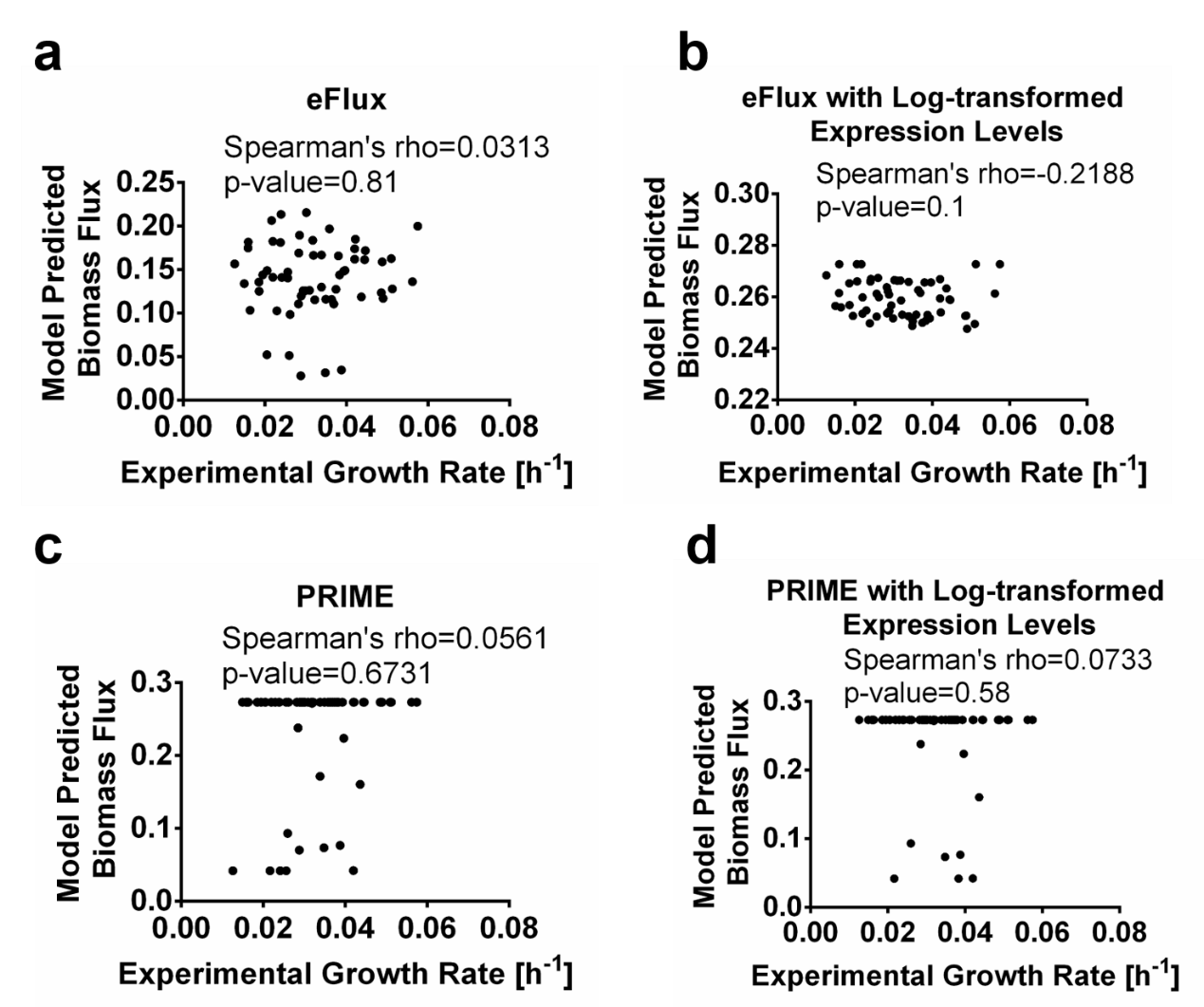


**Figure S2 (Related to Figure 2). Comparisons between actual growth rates of NCI-60 cell lines and growth rates predicted by other methods.**

(a) Comparison between actual growth rates of NCI-60 cell lines and predictions by e-Flux using raw protein expression levels as the input dataset.

(b) Same as in (a) but using log-transformed protein expression levels as input.

(c) Comparison between actual growth rates of NCI-60 cell lines and predictions by PRIME using raw protein expression levels as the input dataset.

(d) Same as in (c) but using log-transformed protein expression levels as input.


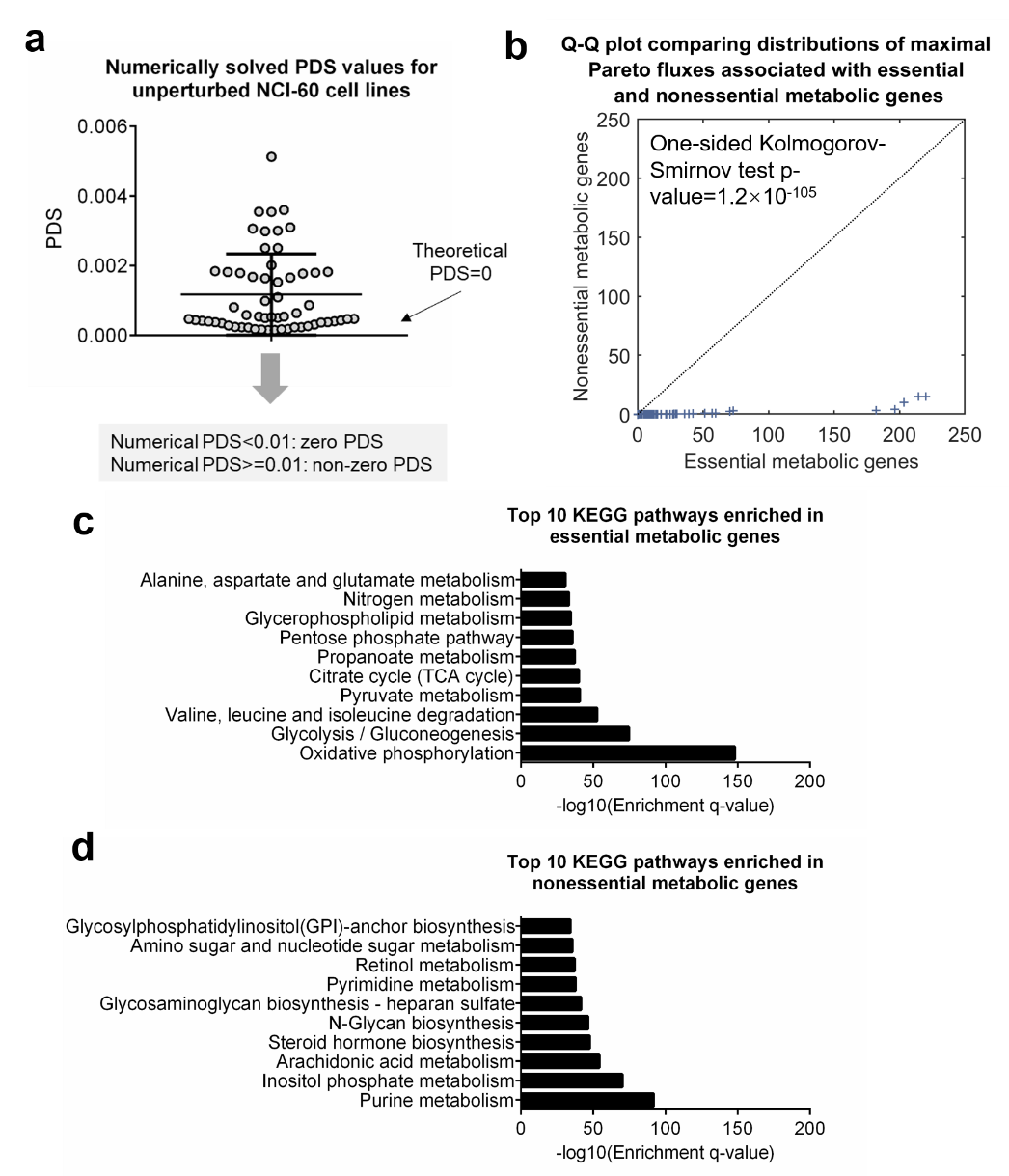


**Figure S3 (Related to Figure 2). Comparison between model-predicted essential and nonessential metabolic genes.**

(a) Numerically computed PDS values for NCI-60 cell lines without perturbations.

(b) Quantile-quantile (Q-Q) plot comparing distributions of maximal Pareto fluxes (i.e. maximal flux carried by the corresponding reaction across all Pareto solutions) associated with essential and nonessential metabolic genes. The p-value was computed using one-sided Kolmogorov-Smirnov test.

(c) Top 10 KEGG metabolic pathways enriched in model-predicted essential metabolic genes.

(d) Top 10 KEGG metabolic pathways enriched in model-predicted nonessential metabolic genes.


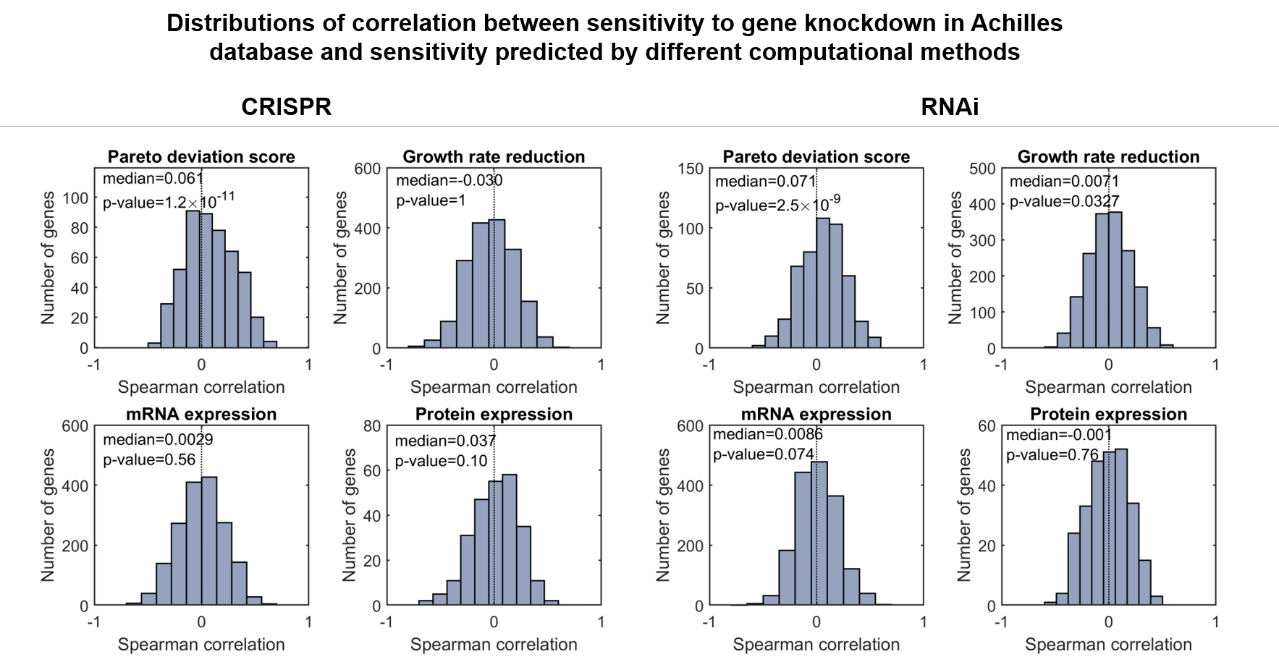


**Figure S4 (Related to Figure 2). Distributions of Spearman’s rank correlation coefficients between experimentally measured sensitivities of NCI-60 cell lines to metabolic gene knockdowns and sensitivities predicted by different computational methods.** P-values were computed using one-sided Wilcoxon’s signed rank test with the null hypothesis of median value = 0. Left panel: CRISPR-based gene ablation dataset; right panel: RNAi-based gene ablation dataset.


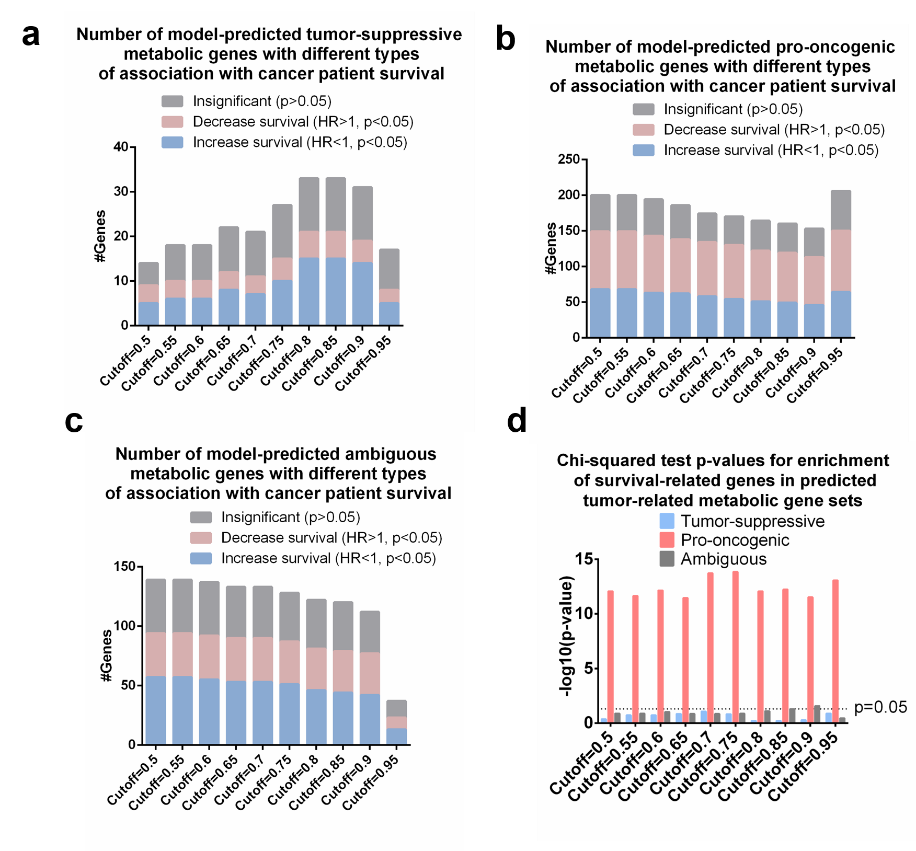


**Figure S5 (Related to Figure 3). Influences of monotonousness score cutoff used in Pareto surface analysis on the associations between model-predicted targets and cancer patient survival.**

(a) Relationship between monotonousness score cutoff used in the Pareto surface analysis and resulting numbers of model-predicted tumor-suppressive metabolic genes associated with decreased, increased or unchanged breast cancer patient survival.

(b) Same as in (a) but for pro-oncogenic metabolic genes.

(c) Same as in (a) but for ambiguous genes.

(d) Relationship between monotonousness score cutoff used in the Pareto surface analysis and chi-squared p-values for enrichment of metabolic genes associated with decreased, increased or unchanged breast cancer patient survival in the resulting pro-oncogenic, tumor-suppressive or ambiguous genes.


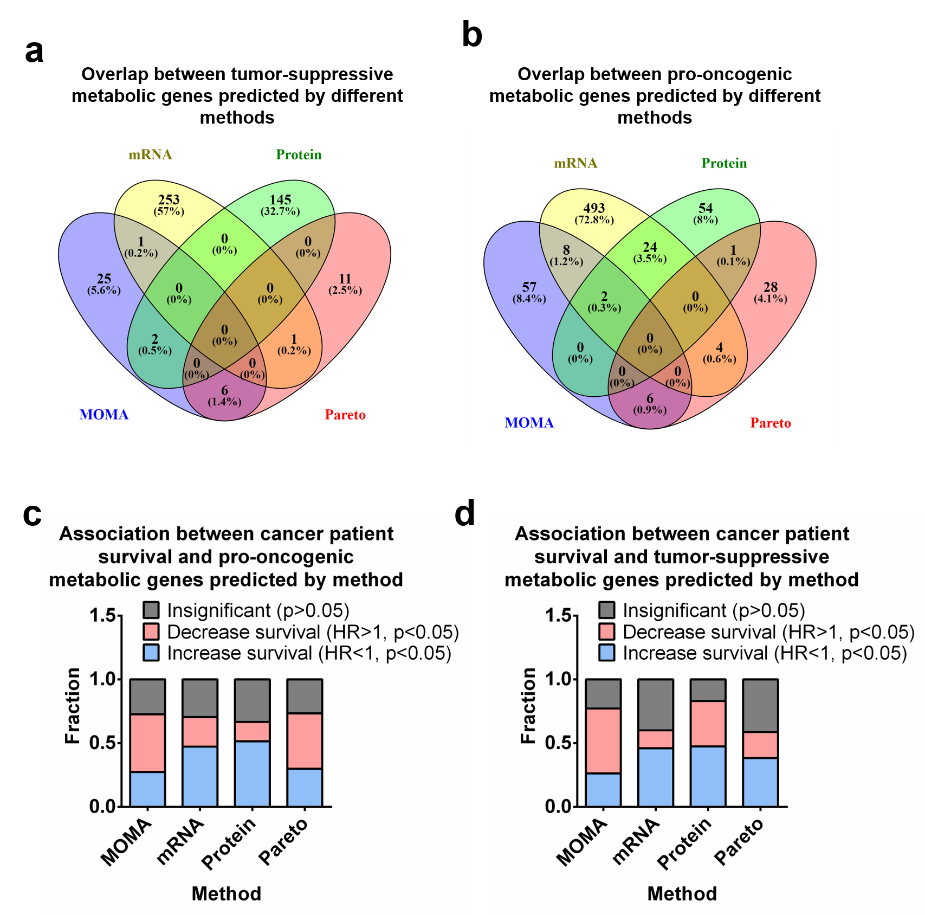


**Figure S6 (Related to Figure 3). Comparison between targets identified by Pareto surface analysis and other methods.**

(a) Venn diagram comparing tumor-suppressive metabolic genes identified by Pareto surface analysis and other methods.

(b) Venn diagram comparing pro-oncogenic metabolic genes identified by Pareto surface analysis and other methods.

(c) Numbers of genes associated with decreased, increased or unchanged breast cancer patient survival in pro-oncogenic genes predicted by Pareto surface analysis and other methods.

(d) Numbers of genes associated with decreased, increased or unchanged breast cancer patient survival in tumor-suppressive genes predicted by Pareto surface analysis and other methods.


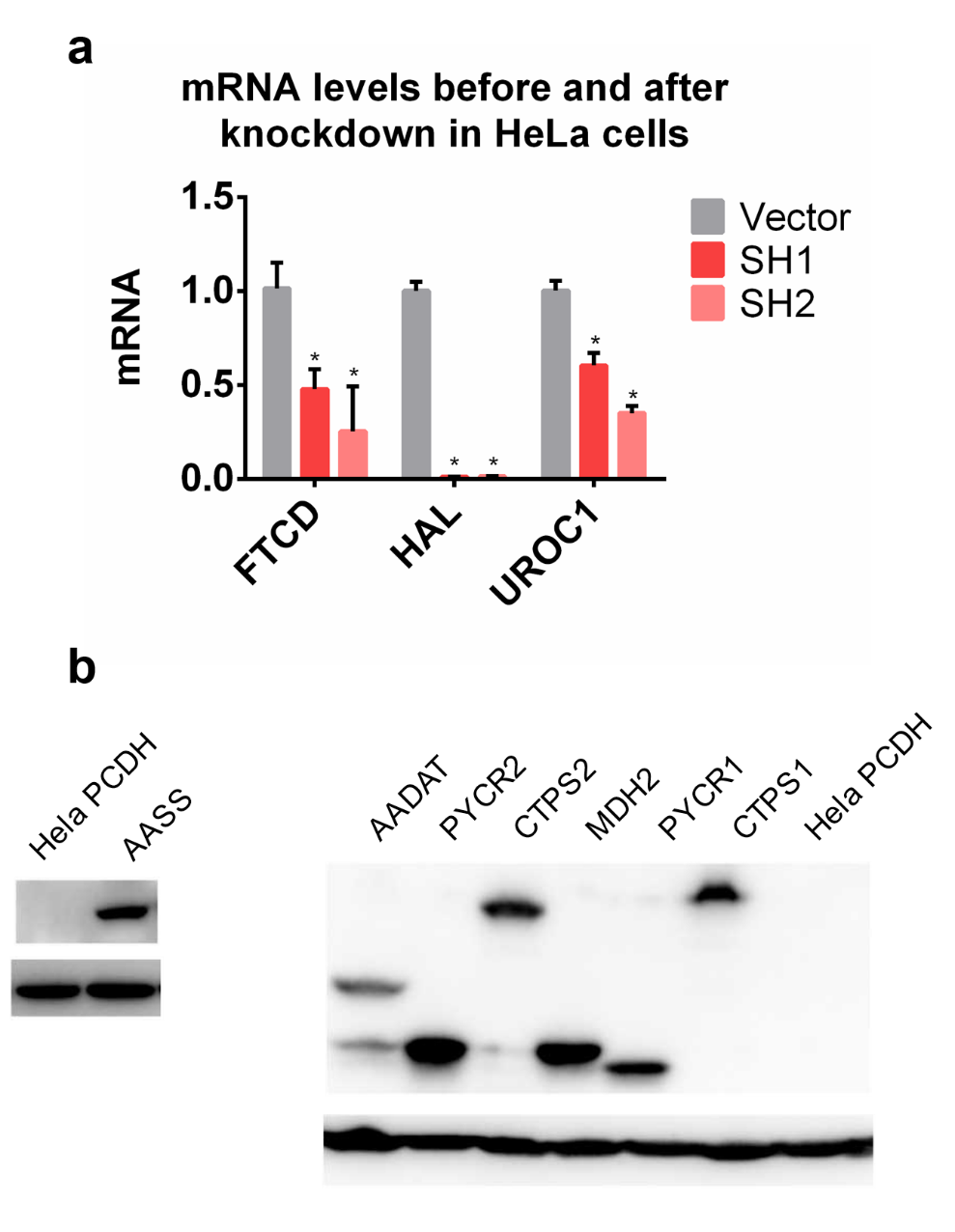


**Figure S7 (Related to Figures 4-6). Validation of efficiencies of gene knockdowns and over-expressions.**

(a) mRNA levels of FTCD, HAL and UROC1 with or without knockdown in HeLa cells.

(b) Protein abundances of AASS, AADAT, PYCR1, PYCR2, CTPS1, CTPS2 and MDH2 with or without over-expressions of the corresponding genes in HeLa cells.


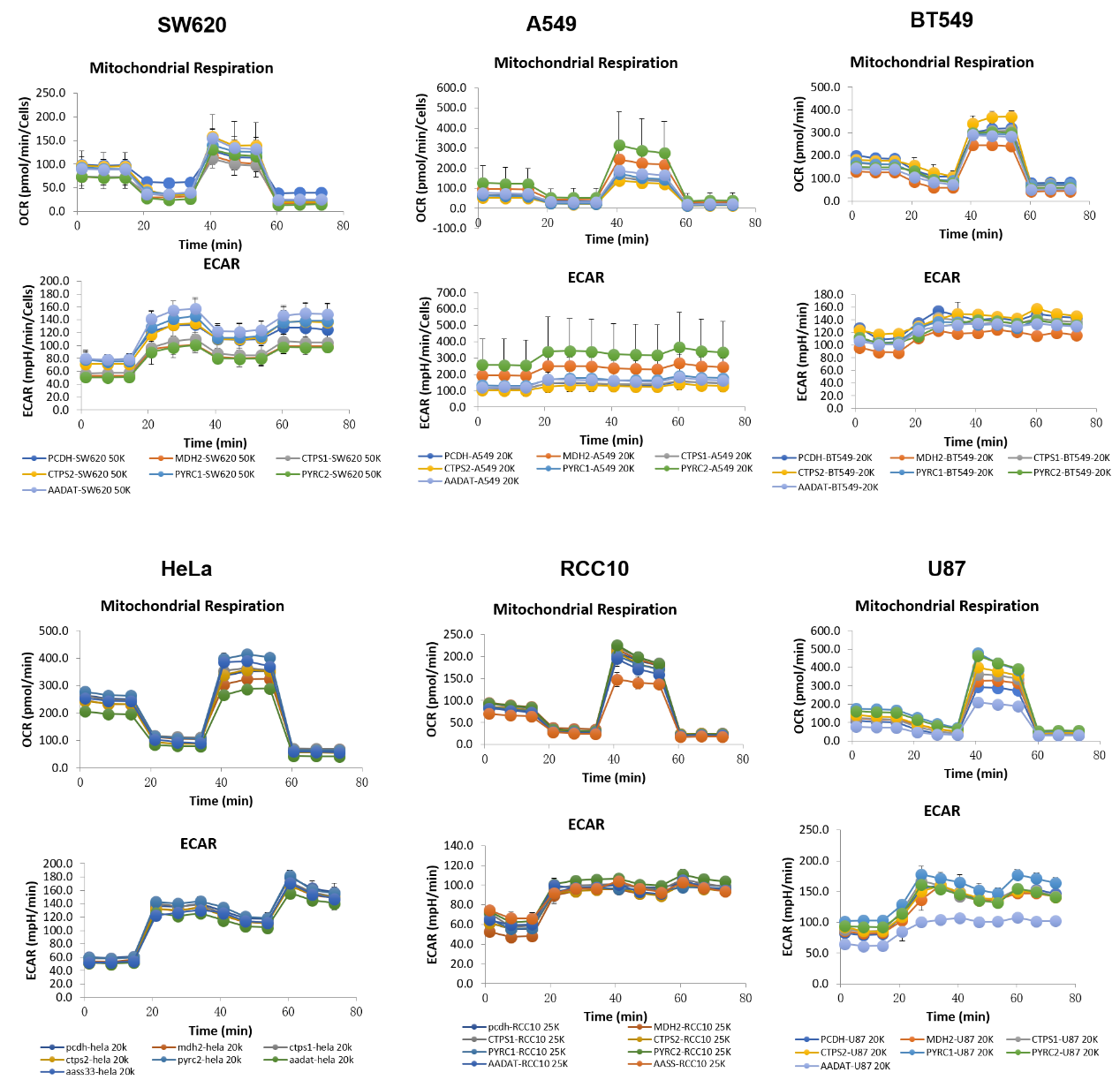


**Figure S8 (Related to Figure 6). Mitochondrial respiration and ECAR profiles of SW620, A549, BT549, HeLa, RCC10 and U87 cells with or without over-expression of MDH2, CTPS1, CTPS2, PYCR1 or PYCR2.**

**
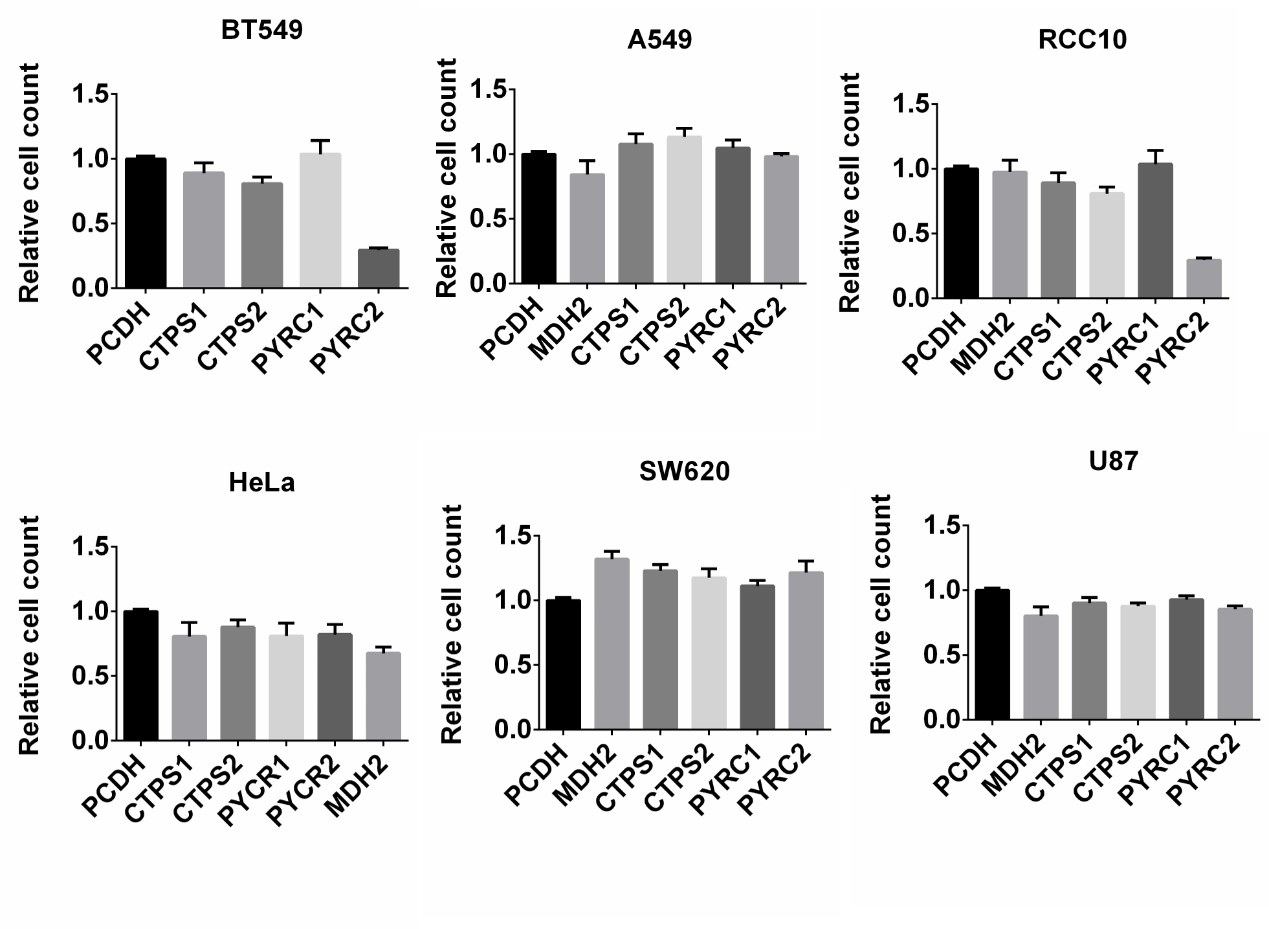
**

**Figure S9 (Related to Figure 6). Relative number of cells after 4 days in the control group (PCDH) or upon over-expression of MDH2, CTPS1, CTPS2, PYRC1 or PYRC2 in the tested cell lines.**

**Supplementary Methods**

**Genome-scale metabolic model and definition of metabolic objectives**

We considered four metabolic objectives including maximization of biomass production flux (f_BM_), maximization of ATP turnover (f_ATP_), minimization of carbon uptake (CU), and minimization of total metabolic enzyme abundance (EA). The genome-scale metabolic model used here, Recon 1, already contains a biomass producing flux whose coefficients are determined by the molecular composition of mammalian cells. We employed a curated and decomposed (i.e. all reversible reactions are decomposed into forward and backward reactions) Recon 1 model by Shlomi *et al* to simplify the following procedures. Model files in the SBML format were downloaded from the BioModels Database (<https://www.ebi.ac.uk/biomodels/>, Model ID: MODEL1105100000). We then translated the SBML files into MATLAB .m files using the COBRA Toolbox^1^, and supplemented the original model with additional biomass components for the following analysis. An ATP hydrolysis flux was directly included in the model. The carbon uptake (CU) flux was calculated as follows:

$$CU=\sum_{i=1}^{n} n_{C,i}f_{i}$$

n is number of fluxes in the model, $n_{C,i}$ is the number of carbon atoms imported into intracellular compartments by the i-th flux and $f_{i}$ is its flux rate. If this flux does not lead to any carbon uptake, the value of $n_{C,i}$ is zero.

The enzyme abundance (EA) was determined by:

$$EA=\sum_{i=1}^{n} \frac{W_{i}}{K_{cat,i}}f_{i}$$

Since the coefficients $\frac{W_{i}}{K_{cat,i}}$ have already been evaluated in the curated model in an artificial flux corresponding to the solvent capacity constraint, these values were directly utilized in our analysis. The artificial solvent capacity flux was removed from the model. Upper limits of nutrient influxes were set according to the composition of RPMI-1640 medium. Details about the model is included in Supplementary Table 1.

**Sampling the Pareto surface**

Pareto solutions of optimization problems with *N* objectives were generated using $\varepsilon$-Constraint Method, in which a single objective selected from the *N* objectives is optimized while the remaining *N*-1 of the objectives are transformed into soft constraints. In general, a multi-objective optimization problem on a genome-scale metabolic model can be written in the form below:

$$\max(f_{1}\left( \boldsymbol{x} \right),f_{2}\left( \boldsymbol{x} \right), \ldots,f_{N}\left( \boldsymbol{x} \right))$$

$$s.t. \boldsymbol{S}\cdot\boldsymbol{x}=0, \boldsymbol{0}\leq\boldsymbol{x}\leq\boldsymbol{ub}$$

In which ***S*** is the stoichiometric matrix for the metabolic network, ***x*** is the flux vector in which each element is the rate of a reaction (*i.e.* a flux), ***ub*** is the vector of maximal rates of reactions. Assume that $f_{i}\left( \boldsymbol{x} \right)$ is the objective to be optimized and all other objectives are treated as constraints, the single-objective optimization problem below, if feasible, gives a solution with Pareto optimality for the original multi-objective optimization problem:

$$\max f_{i}\left( \boldsymbol{x} \right), s.t.$$

$$\left\{ \begin{aligned} f_{1}\left( \boldsymbol{x} \right)\geq e_{1} \\ \ldots\\ f_{i-1}\left( \boldsymbol{x} \right)\geq e_{i-1} \\ f_{i+1}\left( \boldsymbol{x} \right)\geq e_{i+1} \\ \ldots\\ f_{N}\left( \boldsymbol{x} \right)\geq e_{N} \\ \boldsymbol{S}\boldsymbol{\cdot}\boldsymbol{x}\boldsymbol{=0,0\leq}\boldsymbol{x}\boldsymbol{\leq}\boldsymbol{ub} \end{aligned} \right.$$

By selecting different combinations of objectives to be transformed to constraints, adjusting the ε-values (*e_1_, …, e_N_*), and repeatedly solving the corresponding single objective optimization problem, we can obtain a set of solutions with Pareto optimality. In our model all objectives and constraints are linear, thus enabling us to solve the single objective optimization problems with efficient algorithms (e.g. simplex method, interior point method, etc) for linear programming (LP). The LP problems were solved using Mosek (<http://www.mosek.com/>).

To generate feasible linear programming problems more efficiently, we applied a two-step pre-processing strategy to select combinations of ε-values that yield feasible single-objective linear programming problems. First, we calculated the maximal values for the biomass production flux ($f_{Biomass}^{max}$) and the ATP hydrolysis flux ($f_{ATP}^{max}$). We then sampled 10000 combinations of $(e_{Biomass}, e_{ATP})$ in the region $[0, f_{Biomass}^{max}]\times[0, f_{ATP}^{max}]$ with Latin Hypercube Sampling. For each combination we first checked the feasibility of the linear programming problem with the two additional constraints $f_{Biomass}\geq e_{Biomass}$ and $f_{ATP}\geq e_{ATP}$, and then calculated for each feasible $(e_{Biomass}, e_{ATP})$ the feasible ranges of CU and EA ($[{CU}_{min}, {CU}_{max}]$ and $[{EA}_{min}, {EA}_{max}]$) compatible with constraints in the GSMM, $f_{Biomass}\geq e_{Biomass}$ and $f_{ATP}\geq e_{ATP}$. Calculating the feasible range of $[{CU}_{min}, {CU}_{max}]$ and $[{EA}_{min}, {EA}_{max}]$ according to sampled $(e_{Biomass}, e_{ATP})$ prior to generation of the single-objective optimization problems helps avoid generating a large number of infeasible problems. We then applied stratified sampling to select $m_{1}$ values within $[{CU}_{min}, {CU}_{max}]$ and $m_{2}$ values within $[{EA}_{min}, {EA}_{max}]$ and used these randomly sampled values as the upper limits for CU or EA to generate $m_{1}+m_{2}$ LP problems among which $m_{1}$ problems minimize EA and $m_{2}$ minimize CU. The values of $m_{1}$ and $m_{2}$ were determined by the range of $[{CU}_{min}, {CU}_{max}]$ and $[{CU}_{min}, {CU}_{max}]$ with 10 as the maximal value. Solutions of all feasible linear programming problems (42930 in total) were summed up as the sampled Pareto surface.

**Retrieving and processing the omics datasets**

Proteomics data of the NCI-60 cell lines are available at the NCI-60 Proteome Resource: <http://wzw.tum.de/proteomics/nci60>. The exchange flux rates of these cell lines were obtained from a study at 2012^2^. Protein abundances and exchange fluxes were normalized to cell sizes by dividing cell volumes calculated based on cell diameters available at: <http://www.nexcelom.com/Applications/Cancer-Cells.html>, with the assumption that single cells are perfect spheres. Expression levels of metabolic enzymes were evaluated based on proteomics data and gene-protein-reaction rules (GPR rules) included in the GSMM. GPR rules are logical expressions connecting genes, enzymes and reactions. These expressions include logic operators AND, OR and brackets. Expression levels of enzymes were estimated by incorporating the expression levels of genes to the GPR formulas and then evaluating the formulas with AND operators treated as minimum operators and OR operators treated as addition operators. Below is one example of GPR rules:

$$E=R(G_{1}, G_{2},G_{3})=G_{1} AND (G_{2} OR G_{3})$$

In which *R* is the GPR rule connecting the enzyme *E* and $G_{1}$, $G_{2}$ and $G_{3}$ which are three transcripts related to the enzyme in the following way: E has two subunits, one is translated from $G_{1}$, the other can be translated from either $G_{2}$ or $G_{3}$. Let $g_{1}$, $g_{2}$ and $g_{3}$ denote abundance of the three transcripts, the relative abundance of E, e, is evaluated by:

$$e=min(g_{1}, g_{2}+g_{3})$$

CRISPR-based gene dependency dataset in the Achilles database (“gene_dependency_corrected.csv”) was retrieved from <https://figshare.com/articles/DepMap_Achilles_19Q1_Public/7655150>. RNAi-based gene dependency dataset (“D2_Achilles_gene_dep_scored.csv”) in the Achilles database was retrieved from <https://figshare.com/articles/DEMETER2_data/6025238/5>. Cell growth rates were evaluated based on doubling times available at: <http://discover.nci.nih.gov/cellminer/>.

**Construction of cell line-specific models**

We defined a similarity score to evaluate to which extent the distribution of flux configurations predicted by the individualized models can reproduce the corresponding omics data. The similarity metric (S) is comprised of two parts: $S=S_{1}+S_{2}$. $S_{1}$ is the summation of correlation coefficients between model-predicted and experimentally-determined exchange fluxes:

$$S_{1}=\sum_{i=1}^{n_{\mathrm{exc}}} Corr[\left( f_{1,i}^{\exp}, f_{2,i}^{\exp},\ldots f_{n,i}^{\exp} \right), (f_{1,i}^{\mathrm{model}}, f_{2,i}^{\mathrm{model}},\ldots f_{n,i}^{\mathrm{model}})]$$

In which n is the total number of cell lines, $n_{\mathrm{exc}}$ is the total number of experimentally measured exchange fluxes, $f_{k,i}^{\exp}$ is the experimentally measured value of the i-th exchange flux in the k-th cell line, $f_{k,i}^{\mathrm{model}}$ is the model-predicted value of the i-th exchange flux in the k-th cell line.

$S_{2}$ is the summation of correlation coefficients between model-predicted average flux rates and average expression levels of metabolic pathways:

$$S_{2}=\sum_{i=1}^{n_{P}} Corr[\left( e_{1,i}^{\exp}, e_{2,i}^{\exp},\ldots e_{n,i}^{\exp} \right), (f_{1,i}^{\mathrm{model}}, f_{2,i}^{\mathrm{model}},\ldots f_{n,i}^{\mathrm{model}})]$$

In which $n_{P}$ is the total number of metabolic pathways in the KEGG database, $e_{k,i}^{exp}$ is the average protein expression level of metabolic enzymes included in the i-th pathway for the k-th cell line, $f_{k,i}^{\mathrm{model}}$ is the model-predicted average flux rate for reactions included in the i-th pathway for the k-th cell line. Spearman’s rank correlation coefficient was used to quantify the correlation. Single enzymes were mapped to metabolic pathways according to the BRENDA Database based on their EC Numbers. The cell line-specific models (i.e. flux configurations for the cell lines) were constructed by searching for combination of Pareto solutions maximizing the similarity metric S using simulated annealing.

**Calculation of the Pareto deviation score**

We simulated the effects of metabolic gene knockdowns using minimization of metabolic adjustments (MOMA)^3^. Metabolic gene knockdowns were simulated by reducing upper bounds to zero for fluxes carried by enzymes associated with the ablated genes. For a flux configuration $\boldsymbol{x}_{0}$, the new flux configuration after knockdown of the i-th gene was calculated by solving the quadratic programming problem below:

$$\max\left\| \boldsymbol{x}-\boldsymbol{x}_{0} \right\|^{2}, s.t.$$

$$\left\{ \begin{aligned} \boldsymbol{S}\cdot\boldsymbol{x}=\boldsymbol{0} \\ \boldsymbol{x}\geq\boldsymbol{0} \\ \boldsymbol{x}\leq\boldsymbol{ub} \\ x_{j}\leq0, for all \mathrm{reaction} j associated with gene i \end{aligned} \right.$$

Inhibition of glycolysis was simulated by reducing the upper bound of flux carried by the reaction “hexokinase (D-glucose:ATP)” to 10% of the current flux value. Inhibition of OXPHOS was simulated by reducing the upper bound of flux carried by the reaction “ATP synthase (four protons for one ATP)” to 10% of the current flux value. Flux configuration after inhibition of glycolysis or OXPHOS or both pathways was computed using MOMA as described above.

Let $\boldsymbol{x}_{1}$ denote the simulated new flux configuration after metabolic gene knockdown or metabolic enzyme inhibition, the Pareto deviation score (PDS) was then computed as below:

$$PDS(\boldsymbol{x}_{\boldsymbol{1}})={min}_{\boldsymbol{x}\in PS}\left\| \boldsymbol{(}f_{BM}\left( \boldsymbol{x} \right),f_{ATP}\left( \boldsymbol{x} \right),CU\left( \boldsymbol{x} \right),EA\left( \boldsymbol{x} \right))-\boldsymbol{(}f_{BM}\left( \boldsymbol{x}_{\boldsymbol{1}} \right),f_{ATP}\left( \boldsymbol{x}_{\boldsymbol{1}} \right),CU\left( \boldsymbol{x}_{\boldsymbol{1}} \right),EA\left( \boldsymbol{x}_{\boldsymbol{1}} \right)) \right\|$$

In which PS denotes the set containing all sampled Pareto solutions, $f_{BM}\left( \boldsymbol{x} \right)$, $f_{ATP}\left( \boldsymbol{x} \right)$, $CU\left( \boldsymbol{x} \right)$ and $EA\left( \boldsymbol{x} \right)$ are the biomass synthesis flux, ATP hydrolysis flux, carbon uptake and total metabolic enzyme abundance corresponding to flux configuration $\boldsymbol{x}$. PDS values below 0.01 were converted to zero to correct for numerical errors (Supplementary Figure 3a).

**Pareto surface analysis**

The upper and lower bounds of projected Pareto surface were approximated by mathematical discretization. Ranges of cell growth rate or the Warburg effect metric (defined as the ratio of lactate secretion flux to glucose uptake flux) of all Pareto solutions were divided into 50 bins with identical sizes. Let $f_{i}$ denote the variable describing the *i*-th flux, the lower bounds (i.e. minimal values) and upper bounds (i.e. maximal values) of $f_{i}$ in all Pareto solutions whose growth rates or the Warburg effect metric fell in each of the 50 bins were calculated as $[{LB}_{1}^{i},{LB}_{2}^{i},\ldots,{LB}_{50}^{i}]$ and $[{UB}_{1}^{i},{UB}_{2}^{i},\ldots,{UB}_{50}^{i}]$. The monotonous scores for upper and lower bounds of this flux was quantified by Spearman’s rank correlation coefficients between the vector $[{LB}_{1}^{i},{LB}_{2}^{i},\ldots,{LB}_{50}^{i}]$ or $[{UB}_{1}^{i},{UB}_{2}^{i},\ldots,{UB}_{50}^{i}]$ and the vector [1, 2, ..., 50]. Bounds were considered as monotonously decreasing if the monotonous scores were less than -0.9, and monotonously increasing if larger than 0.9.

**Patient survival and pathway enrichment analysis**

Kaplan-Meier survival analyses were performed using the Kaplan-Meier Plotter (<http://kmplot.com/analysis/>)^4^ using the probe set option ‘only JetSet best probe set’, patients split option ‘Auto select best cutoff’. Default parameters were used for all other options not mentioned. For genes without JetSet best probe sets, the probe set with maximal number of patients was selected. Pathway enrichment analysis was performed using the module ‘Investigate Gene Sets’ available at the Molecular Signatures Database (MSigDB, <http://software.broadinstitute.org/gsea/msigdb/index.jsp>).

**Comparison with alternative methods**

For construction of cell line-specific models, PRIME^5^ and E-Flux^6^ methods were implemented in MATLAB codes using proteomics data for NCI-60 cell lines as the input. Raw intensity values were either directly used (Supplementary Fig. 2a, c) or log-transformed (Supplementary Fig. 2b, d) using the expression $y=\log(x+1)$ before being used as input of the PRIME and E-Flux algorithms. Doubling times of the NCI-60 cell lines were also used as the input for PRIME.

For identification of putative metabolic targets, three alternative methods including correlation between mRNA abundances and growth rates, correlation between protein expression levels and growth rates and minimization of metabolic adjustments (MOMA) were applied. For the correlation-based methods, correlations between NCI-60 cancer cell proliferation and mRNA or protein expression levels of metabolic enzymes in these cell lines were quantified using Spearman’s rank correlation coefficients. Metabolic enzymes with mRNA or protein expression level significantly correlated with growth rates of NCI-60 cell lines (i.e. Benjamini-Hochberg adjusted p-value<0.05) were identified as tumor-suppressive (Spearman’s correlation coefficient<0) or tumor-promoting (Spearman’s correlation coefficient>0) targets. For the MOMA-based method, MOMA was applied to simulate the effects of inhibition or activation of metabolic enzymes on growth rates of NCI-60 cell lines. Inhibition of a metabolic enzyme in a cell line-specific model was simulated by reducing the upper bound of the flux catalyzed by this enzyme to 10% of the current value of this flux in the corresponding model, while activation of a metabolic enzyme was simulated by adjusting the lower bound of the flux catalyzed by this enzyme to two-folds of the current flux rate. MOMA was then applied to compute the new metabolic flux configuration upon the inhibition or activation. Average relative reduction of the biomass synthesis flux in the NCI-60 cell line-specific models was used as the metric for target identification. Metabolic enzymes whose inhibition or activation resulted in more than 50% relative reduction of biomass synthesis flux averaged over the NCI-60 cell line models were identified as tumor-promoting or tumor-suppressing targets.

**References**

1 Schellenberger, J. *et al.* Quantitative prediction of cellular metabolism with constraint-based models: the COBRA Toolbox v2.0. *Nat Protoc* **6**, 1290-1307, doi:10.1038/nprot.2011.308 (2011).

2 Jain, M. *et al.* Metabolite profiling identifies a key role for glycine in rapid cancer cell proliferation. *Science* **336**, 1040-1044, doi:10.1126/science.1218595 (2012).

3 Segre, D., Vitkup, D. & Church, G. M. Analysis of optimality in natural and perturbed metabolic networks. *Proc Natl Acad Sci U S A* **99**, 15112-15117, doi:10.1073/pnas.232349399 (2002).

4 Szasz, A. M. *et al.* Cross-validation of survival associated biomarkers in gastric cancer using transcriptomic data of 1,065 patients. *Oncotarget* **7**, 49322-49333, doi:10.18632/oncotarget.10337 (2016).

5 Yizhak, K. *et al.* Phenotype-based cell-specific metabolic modeling reveals metabolic liabilities of cancer. *Elife* **3**, doi:10.7554/eLife.03641 (2014).

6 Colijn, C. *et al.* Interpreting expression data with metabolic flux models: predicting Mycobacterium tuberculosis mycolic acid production. *PLoS Comput Biol* **5**, e1000489, doi:10.1371/journal.pcbi.1000489 (2009).
